# VPS72/YL1-Mediated H2A.Z Deposition Is Required for Nuclear Reassembly after Mitosis

**DOI:** 10.3390/cells9071702

**Published:** 2020-07-16

**Authors:** Daniel Moreno-Andrés, Hideki Yokoyama, Anja Scheufen, Guillaume Holzer, Hongqi Lue, Anna Katharina Schellhaus, Marion Weberruss, Masatoshi Takagi, Wolfram Antonin

**Affiliations:** 1 Institute of Biochemistry and Molecular Cell Biology, Medical School, RWTH Aachen University, 52074 Aachen, Germany; hideki-yokoyama@idpharma.jp (H.Y.); anscheufen@ukaachen.de (A.S.); gholzer@ukaachen.de (G.H.); hlue@ukaachen.de (H.L.); katharina.schellhaus@ki.se (A.K.S.); marion.weberruss@gmx.de (M.W.); 2 Friedrich Miescher Laboratory of the Max Planck Society, Spemannstrasse 39, 72076 Tübingen, Germany; 3 Cellular Dynamics Laboratory, RIKEN Cluster for Pioneering Research, 2-1 Hirosowa, Wako, Saitama 351-0198, Japan; mtakagi@riken.jp

**Keywords:** VPS72, YL-1, vacuolar protein sorting 72 homolog, YL1, CFL1, Swc2, TCFL1, H2A.Z, H2AZ, nuclear envelope, nucleolus, mitotic exit, telophase, nuclear reformation

## Abstract

The eukaryotic nucleus remodels extensively during mitosis. Upon mitotic entry, the nuclear envelope breaks down and chromosomes condense into rod-shaped bodies, which are captured by the spindle apparatus and segregated during anaphase. Through telophase, chromosomes decondense and the nuclear envelope reassembles, leading to a functional interphase nucleus. While the molecular processes occurring in early mitosis are intensively investigated, our knowledge about molecular mechanisms of nuclear reassembly is rather limited. Using cell free and cellular assays, we identify the histone variant H2A.Z and its chaperone VPS72/YL1 as important factors for reassembly of a functional nucleus after mitosis. Live-cell imaging shows that siRNA-mediated downregulation of VPS72 extends the telophase in HeLa cells. In vitro, depletion of VPS72 or H2A.Z results in malformed and nonfunctional nuclei. VPS72 is part of two chromatin-remodeling complexes, SRCAP and EP400. Dissecting the mechanism of nuclear reformation using cell-free assays, we, however, show that VPS72 functions outside of the SRCAP and EP400 remodeling complexes to deposit H2A.Z, which in turn is crucial for formation of a functional nucleus.

## 1. Introduction

The eukaryotic cell nucleus reorganizes considerably during cell division [1,2]. During early mitosis in metazoans, the nuclear envelope breaks down and the chromatin condenses into individualized rod-shaped chromosomes. Then, the spindle apparatus assembles and captures the chromosomes to segregate the sister chromatids during early anaphase. Subsequently, during late anaphase and telophase, the highly condensed mitotic chromosomes decondense, reestablishing a fully functional interphase nucleus. While key molecular processes directing the progression through early mitosis and chromatin segregation have been relatively well investigated, much less is known about those molecular mechanisms mediating de-condensation of mitotic chromosomes and subsequent nuclear reassembly including nuclear envelope reformation [3,4].

Upon mitotic exit, several phosphatases promote the inactivation of mitotic kinases of the Aurora family, polo-like kinase 1 (PLK1) and the cyclin dependent kinase 1 (CDK1) [5,6]. They also mediate massive dephosphorylation of the targets of these kinases, e.g., lamins, nucleoporins and nuclear envelope proteins [7,8,9,10], to enable reassembly of the nuclear envelope and nuclear pore complexes on the new daughter nuclei [11,12]. This allows chromosome segregation during early anaphase and subsequent chromatin de-condensation and nuclear reassembly through late anaphase and telophase. In these last steps, the combined action of the trimeric protein phosphatase 2A (PP2A), defined by its regulatory B55α subunit, and importin-β1 is crucial in human cells [13]. Their presence is necessary for mitotic exit events such as disassembly of the spindle-pole associated microtubules, reestablishing functional nucleocytoplasmic transport, re-clustering of the Golgi apparatus and chromatin de-condensation. Similarly, the protein phosphatase PP1 is involved in reestablishment of an interphase nucleus via its recruitment factor Repo-Man [14,15]. However, the specific molecular mechanisms leading to the successful reformation of interphase nuclei, especially the changes on the chromatin, remain to be clarified.

Recent evidence obtained by dissecting mitotic nuclear reformation using cell-free assays, where *Xenopus laevis* egg extracts were applied on different chromatin templates, suggests that mitotic chromatin de-condensation and nuclear reassembly are multistep processes that influence each other at different levels [16,17,18,19,20]. In this regard, the RuvB-like ATPases pontin and reptin were identified as important mitotic chromatin de-condensation factors [21] using a combination of *Xenopus* sperm nuclear assembly [22] and a newly developed in vitro chromatin de-condensation assay. De-condensation of mitotic chromatin requires ATP and GTP hydrolysis, which can be at least in part explained by the requirement of the ATPases pontin and reptin. However, pontin and reptin are necessary but not sufficient to drive de-condensation of mitotic chromatin. Pontin and reptin re-localize on chromatin of living cells during late anaphase and telophase, consistent with a function in chromatin de-condensation [21,23]. Interestingly, pontin and reptin depletions do not affect nuclear envelope or nuclear pore complex reassembly on *Xenopus* sperm chromatin [21], where sperm DNA decondenses by nucleophosmin-mediated replacement of protamines to histones H2A and H2B [24]. This indicates that pontin and reptin have specific functions in mitotic chromatin de-condensation, which can be, at least in vitro, separated from the reformation of a nuclear envelope and nuclear pore complexes.

As pontin and reptin are ATPases involved in a wide range of cellular activities but are also part of several chromatin remodeling complexes [25,26,27], these results might indicate that chromatin requires energy-dependent histone rearrangements and modifications in order to exit its mitotic state and to decondense. Indeed, the histone demethylase LSD1 (also known as KDM1A) is involved in reformation of the interphase nuclear architecture after mitosis [28]. Upon downregulation of LSD1, human cells showed extended telophase, ectopic nuclear pore complex assembly (annulate lamellae) and smaller interphase nuclei. In the absence of LSD1, mitotic chromatin decondenses only into small, dense and round nuclei. Intriguingly, these nuclei were qualitatively different from the highly compact and irregular, prophase-like structures found in the absence of pontin and reptin [21]. In addition, the lack of LSD1 led to defective nuclear envelope and nuclear pore complex reassembly in *Xenopus* sperm nuclear assembly assays. All this suggests that, while pontin and reptin might work together with other factors early in mitotic chromosome de-condensation, LSD1 could act downstream in order to generate a chromatin template competent for nuclear envelope and nuclear pore complex assembly.

To shed light on the molecular mechanisms that support the action of pontin and reptin during mitotic chromatin de-condensation, we investigated pontin/reptin interacting proteins found in chromatin remodeling complexes that could act as cofactors in chromatin de-condensation. We show here that downregulation of VPS72, also known as YL-1, YL1 or Swc2, extends telophase in cells during mitotic exit similar to pontin and reptin downregulation. VPS72 is, together with pontin and reptin, part of the EP400 and Snf2-related CBP-activator protein (SRCAP) chromatin remodeling complexes. In these complexes, VPS72 functions as a chaperon for H2A to H2A.Z exchange and as a reader for H2A.Z in the ATP-dependent SRCAP or EP400 complexes [29,30]. Our results indicate that VPS72 functions in nuclear reassembly as an H2A.Z chaperon but independent of SRCAP and EP400. Depletion of not only VPS72 but also H2A.Z impairs chromatin structure as well as compactness and results in malformed nuclear envelopes.

## 2. Materials and Methods

### 2.1. Recombinant Proteins and Antibodies

*Xenopus laevis* VPS72 as well as fragments of *Xenopus tropicalis* Ino80 (aa 195-403), EP400 (aa 1-347), SRCAP (aa 1-311) and *Xenopus laevis* nucleolin (aa 229-651) were cloned as codon-optimized sequences for expression in *E. coli* into a modified pET28a vector with a yeast small ubiquitin-like modifier (SUMO) solubility tag, which is followed by a Tobacco Etch Virus (TEV) cleavage site. *Xenopus laevis* HP1α was cloned as codon-optimized sequences for expression in *E. coli* into pET28a vector. The proteins were expressed in BL21 (DE3) cells, were purified with Ni-NTA-Agarose and were used for antibody production in rabbits. The deletion and point mutants of VPS72 were created by PCR, subcloned into the same modified pET28a vector and purified as above. VPS72, nucleolin and HP1α antisera were affinity purified and used at 3 µg/mL for immunofluorescence and at 1 µg/mL for western blotting. A Western blot further characterizing the Xenopus VPS72 antibody is shown in Figure 3A; please note the absence of the VPS72 signal after depletion, showing the specificity. Ino80, EP400 and SRCAP antisera were used 1:1000 for western blotting. *Xenopus* KI-67 antibody was raised in rabbits against the GST-fusion of the *C*-terminal portion (221 residues) of *Xenopus* KI-67 (XP_018080658.1) and was affinity purified.

The following antibodies were described before: *Xenopus laevis* RuvBL1, RuvBL2 and XCAP-G for western blotting in a 1:1000 dilution [21] and lamin B using 1:100 for immunofluorescence [31]. The following antibodies are commercially available: human β-actin (Sigma, St. Louis, MO, USA, A5441, used 1:5000), human pontin (Sigma, SAB4200194, 1:1000) and reptin (Sigma, SAB4200115, 1:1000), human VPS72 (Biomol, Hamburg, Germany, A303-115A-M, 1:1000), human histone H2B antibody (Merckmillipore, Darmstadt, Germany, 07-371, 1:1000), human H2A.Z (NEB, Danvers, MA, USA, 2718, 1:1000), human Ino80 (Abcam, Berlin, Germany. ab118787, 1:1000), human EP400 (Abcam, ab70301, 1:1000) and human SRCAP (Abcam, ab99408, 1:1000) for western blotting. Human KI-67 (from Millipore, Mab4190, 1:100 for immunofluorescence), H2A.Z (from Millipore 07-594, at 1:500 for immunofluorescence and at 1:1000 for western blotting), H3K27me3 (from Cell Signaling, Danvers, MA, USA, 9728P, 1:1000 for immunofluorescence), H3K3me3 (from Abcam, 8580, 1:1000 for immunofluorescence), H3K9me3 (from Merck, Darmstadt, Germany, 07-523, 1:1000 for immunofluorescence), SUMO (Boston Biochemicals, Cambridge, MA, USA A-722, 1:1000 for western blotting) and mAb414 (from Covance, MMS-120R, 1:2000 for immunofluorescence). Secondary antibodies for immunofluorescence were Alexa-Fluor-488-anti-mouse and Alexa-Fluor-547-anti-rabbit (from Life technologies, Carlsbad, CA, USA, 1:1000).

### 2.2. Chromatin Re-Isolation

Cytostatic factor (CSF)-arrested *Xenopus* egg extract was prepared as described previously [32]. The extract was incubated in the presence or absence of 1250 sperm/µL and 0.4 mM CaCl_2_. At each time point, an aliquot was taken, diluted with 10 volumes of a dilution buffer (10 mM K-HEPES, 50 mM KCl, 5 mM MgCl_2_, 2 mM DTT, 0.5 mM spermidine, 0.15 mM spermine, 2.5 mM ATP and 0.1% Triton X-100) and centrifuged at 5000× *g* for 5 min through a 15% sucrose cushion in the same buffer. After removing the majority of the supernatant, chromatin pellets were washed with the dilution buffer 3 times and centrifuged at 10,000× *g* for 2 min. The supernatant was removed completely, and the chromatin fraction was recovered for Western blots.

### 2.3. Nuclear Assembly Assay and Chromatin De-Condensation Assay

Interphase *Xenopus* egg extracts were prepared as described previously [21,33]. For depletion, the extract was incubated twice with rabbit IgG (as control) or *Xenopus* VPS72 antibody-coated beads at a 1.4:1 bead-to-extract ratio for 30 min each.

For sperm nuclear assembly, 20 µL extracts were incubated with 3000 sperm heads at 20 °C for 10 min. The reactions were supplemented with energy mix (10 mM ATP, 10 mM GTP, 10 mM creatine phosphate and 0.2 mg/mL creatine kinase), 0.4 mg/mL glycogen and 2 µL of flotation-purified membranes and incubated for 2 h. The samples were fixed with 2% paraformaldehyde and 0.5% glutaraldehyde in 80 mM PIPES, 1 mM MgCl_2_, 150 mM sucrose and 10 µg/mL DAPI (4,6-Diamidin-2-phenylindol) for 20 min on ice. Nuclei were centrifuged through a 30% sucrose cushion in PBS (15 min at 2500× *g*) on poly-l-lysine-coated coverslips and mounted in Mowiol. To immunostain the nuclei, glutaraldehyde was omitted from the fixation buffer. To deplete H2A.Z from egg extracts, recombinant VPS72 aa1-97 was immobilized to Affi-Gel 10 (Bio-Rad, Hercules, CA, USA) according to the manufacture’s instruction. The extract was incubated twice with control or aa 1-97 beads at a 1.4:1 bead-to-extract ratio each time for 30 min. Recombinant human histone H2A.Z-H2B dimer (Millipore, Darmstadt, Germany) was added to the nuclear assembly at 0.3 µM. To monitor DNA replication, the egg extract was supplemented with 5 µM Cy3-labeled dUTP (Roche, Basel, Switzerland) and used for nuclear assembly [32].

For the chromatin de-condensation assay, mitotic chromatin clusters were isolated from HeLa cells [34]). As described in Magalska et al. 2014 [21], the 18 µL extract was incubated with 1000 chromatin clusters, 3 µM 6-dimethylaminopurine, 0.4 mg/mL glycogen and the energy mix at 20 °C for 2 h. The samples were fixed with 4% paraformaldehyde and 0.5% glutaraldehyde in 80 mM PIPES, 1 mM MgCl_2_, 150 mM sucrose and 10 µg/mL DAPI for 20 min on ice. Chromatin structures were centrifuged and mounted, as described above in the sperm assay. To visualize the space inside the chromatin, the chromatin was assembled for 2 h. The samples were then supplemented with 0.25 mg/mL of FITC-labeled 2000 kD dextrans and analyzed by confocal microscopy without fixation. To assemble the nuclear envelope around the decondensed chromatin, 2 µL of the floated membranes was supplemented in the assay from the beginning.

Fluorescence microscopy images are recorded on the SP8 confocal microscope (Leica, Wetzlar, Germany) equipped with 63× oil objective and 488-nm and 561-nm lasers using 1 AU pinhole or an Zeiss LSM710 confocal microscope equipped with a Plan-Apochromat 63×/1.4 Oil objective and 405-nm, 488-nm, 561-nm and 633-nm lasers using 1 AU pinhole using ZEN software. Nuclear size was quantified manually using ImageJ. H2A.Z intensity was quantified as in [32].

### 2.4. Cell Culture and Transfection

HeLa cells were maintained in Dulbecco’s modified Eagle’s medium (DMEM) supplemented with 2 mM l-glutamine, 10% fetal bovine serum (FBS) and 500 units/mL penicillin-streptomycin (all from Gibco). HeLa H2B-mCherry/EGFP-IBB and HeLa H2B-mCherry/EGFP-Lap2β cell lines (kind gift from Daniel Gerlich) were additionally supplemented with 0.5 µg/mL puromycin (Gibco) and 500 µg/mL G-418 (Geneticin; Life Technologies, Carlsbad, CA, USA) as described [13]. Additional HeLa cells stably expressing H2B-mCherry were generated as in Schooley et al. 2015 [28] and maintained in DMEM supplemented with 0.5 µg/mL puromycin (Gibco). siRNA oligonucleotides were employed in downregulation experiments (Table 1). Reverse transfections of 20 nM siRNA for life-cell image or 40 nM siRNA for immunofluorescence were carried out in HeLa cell suspensions using lipofectamine RNAiMAX (Invitrogen, Carlsbad, CA, USA) according to the manufacturer’s instructions. Then, the HeLa cells were subjected to live cell image or were processed for immunofluorescence experiments at the indicated times.

### 2.5. Immunofluorescence

siRNA-downregulated HeLa cells expressing H2B-mCherry were processed for immunofluorescence 72 h post-transfection by fixation with 4% paraformaldehyde in PBS for 10 min. Samples were incubated for 2 h at room temperature with the indicated primary antibodies in PBS + 3% bovine fetal serum (BSA) followed by the secondary antibodies. Best in-focus slices from 10 random fields of cells from two replicates in two independent experiments per condition were imaged on a Zeiss LSM710 confocal microscope equipped with a Plan-Apochromat 63×/1.4 Oil objective and 488-nm, 561-nm and 633-nm lasers using 1 AU pinhole using ZEN software.

The quantitation of nuclear intensity and peripheral distribution of the different histone modifications was performed with custom analysis pipelines for CellProfiler [35]. Shortly, nuclei were segmented in base to their H2B-mCherry signal and this region of interest (ROI) was used to define three additional ROIs: chromatin periphery (shrink of 20 px from nuclear ROI), cytoplasm background (expansion of 5 to 20 px of nuclear ROI) and nuclear envelope (toroidal region formed by the expansion of 5 px and shrink of 5 px from nuclear ROI). For each marker per cell, nuclear intensity was calculated as the mean intensity of nuclear ROI minus mean intensity of cytoplasm background and the radial distribution as the mean intensity at the chromatin periphery divided by mean nuclear intensity. For the nuclear pore complex marker (mAB414), the nuclear envelope ROI intensity was calculated as the mean intensity of NE ROI minus mean intensity of cytoplasm background. The peripheral distribution was calculated as the mean intensity at chromatin periphery divided by mean nuclear intensity. Additionally, a machine-learning-based segmentation was performed using Ilastik [36] in the base to the KI-67 marker to generate binary masks of nucleoli. Then, to quantitate accurately number, size and intensity of the nucleoli in each cell, the binary masks were applied to define nucleoli ROIs on the images by mean of custom CellProfiler analysis pipelines.

One representative experiment for each marker is shown. CellProfiller outputs were organized with Microsoft Excel, and GraphPad Prism was used for plotting and statistical analysis. The data were tested for normality using D’Agostino and Pearson omnibus normality test. When normal distributions could not be assumed, statistical significance at alpha = 0.001 was determined using a Kruskal–Wallis test followed by Dunn´s multiple comparisons test. For normal distributions, ANOVA test followed by Dunnett multiple comparisons test was applied.

### 2.6. Nucleolar Function Assays

HeLa cells expressing mCherry-H2B were transfected with 40 nM control or VPS72 siRNA oligos for 72 h. To observe nucleolar integrity living cells were stained with NUCLEOLAR-ID^®^ Green Detection Kit (Enzo, Lausen, Switzerland) in DMEM following manufacture instructions. As a control, cells were treated with 10 µg/mL actinomycin D for 3 h to induce nucleolar segregation. Images were taken with a Ti2 Eclipse microscope (Nikon, Melville, NY, U.S.A.) equipped with an X-light spinning disk, a LED light engine SpectraX (Lumecor, Beaverton, OR, USA), GFP/mCherry filter sets and a Plan-Apochromat 60× NA 1.4 objective. The newly synthetized RNA was visualized by 5-ethynyl uridine incorporation using Click-iT^®^ RNA Imaging Kit (Invitrogen) following manufacture instructions. Shortly, cells were incubated in complete DMEM medium containing 1 mM of 5-ethynyl uridine for 30 min and fixed with 4% paraformaldehyde. After permeabilization with PBS containing 0.5% Triton X-100, the cells were incubated for 30 min in click-chemistry reaction mix containing Alexa Fluor 488 and were mounted for confocal microscopy with Mowiol. As a control, cells were treated with 50 ng/mL actinomycin D for 30 min before 5-ethynyl uridine addition. Five random fields of cells from two independent experiments per condition were imaged on a Zeiss LSM710 confocal microscope equipped with a Plan-Apochromat 63×/1.4 Oil objective and 488-nm, 561-nm and 633-nm lasers with 1 AU pinhole using ZEN software. Quantitation of newly synthetized RNA in the nuclear compartment and the ratio of nucleolus vs. total nuclear signal was performed using the CellProfiler analysis pipelines described above. In this case, the binary masks for nucleoli segmentation and measurement were generated manually using Fiji. Shortly, as nucleoli can be visualized as holes in mCherry-H2B images, nucleoli masks were obtained after applying to all chromatin images the following Fiji tools in row: 8 bit conversion, gaussian blur (1.5), auto-threshold Otsu, LUT inversion and analyze particle (with mask generation). CellProfiller outputs were organized with Microsoft Excel and GraphPad Prism was used for plotting and statistical analysis as described above.

### 2.7. Live-Cell Imaging

**Figure 1:**
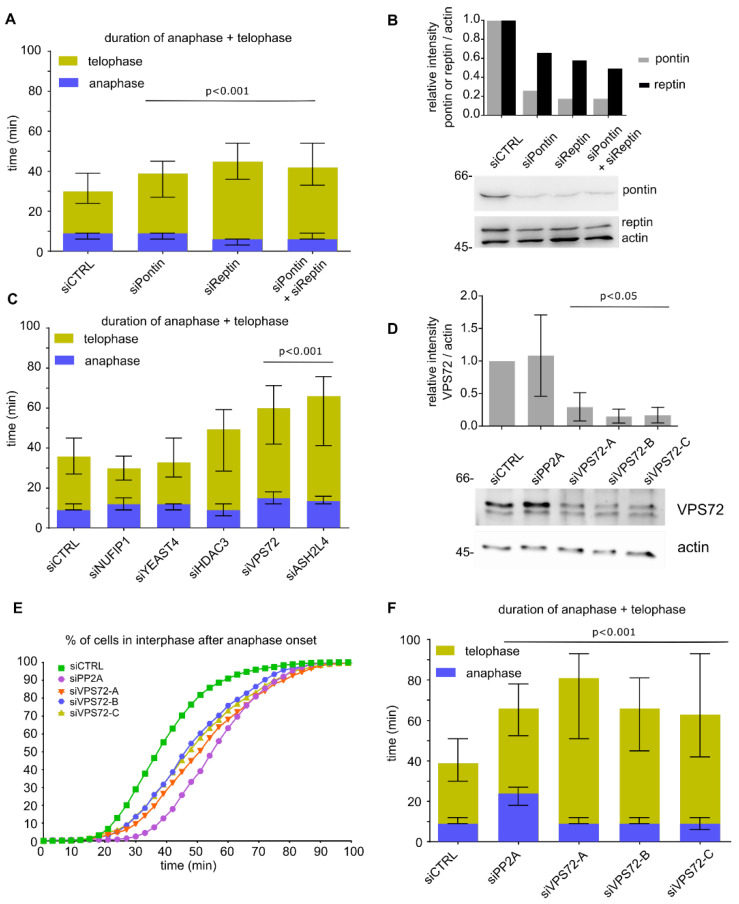
VPS72 downregulation extends telophase: (**A**) HeLa cells expressing mCherry-H2B and EGFP-IBB (importin β binding domain of importin α as a nuclear import substrate) were transfected with 20 nM siRNA oligos against pontin, reptin or both proteins or with control oligos; 30–72 h after transfection, cells were analyzed by life cell imaging and the length of mitotic exit (time from metaphase to anaphase transition until end of telophase) was determined. Length of anaphase (blue) and telophase (yellow) is indicated. The median time spent in late anaphase and telophase is shown for mitotic events from 2 independent experiments. Bars represent interquartile range. Statistical significance at alpha = 0.001 was determined using a Kruskal–Wallis test followed by Dunn´s multiple comparisons test. (**B**) Western blot and quantitation showing the downregulation of pontin and reptin at 55 h post-transfection with 20 nM siRNA oligos in HeLa cells expressing mCherry-H2B and EGFP-IBB. (**C**) HeLa cells expressing mCherry-H2B and EGFP-IBB were transfected with 20 nM siRNA oligos against the indicated pontin/reptin interactors or control oligos; 30–72 h after transfection, cells were analyzed by life cell imaging and the length of mitotic exit (time from metaphase to anaphase transition until end of telophase) was determined. The median time spent in late anaphase and telophase is shown for mitotic events from 2 independent experiments. Colored bars indicate medians, and error bars represent interquartile range. Statistical significance at alpha = 0.001 was determined using a Kruskal–Wallis test followed by Dunn´s multiple comparisons test. The significance values for the candidates are NUFIP1 (*p* = 0.0029), YEAST4 (*p* > 0.9), HDAC3 (*p* = 0.03), VPS72 (*p* < 0.0001) and ASH2L4 (*p* < 0.0001). (**D**) Western blot showing the downregulation of VPS72 using an antibody against the human protein at 48 h post-transfection with 20 nM siRNA oligos in HeLa cells expressing mCherry-H2B. Quantitation is based on three independent experiments. Error bars represent SD; statistical significance was determined by two-tailed student’s test. (**E**) HeLa cells expressing mCherry-H2B were transfected with 20 nM siRNA oligos (negative control, PP2A as positive control and three oligos against VPS72); 48–96 h after transfection, cells were analyzed by life cell imaging and the cumulative percentage of cells in interphase after anaphase onset (*t* = 0 min) was calculated. The curves represent mitotic events from two independent experiments. (**F**) The median time spent in late anaphase and telophase is shown for mitotic events analyzed in (**E**). Bars represent interquartile range. Statistical significance at alpha = 0.001 was determined using a Kruskal–Wallis test followed by Dunn´s multiple comparisons test.

For live imaging experiments, the indicated HeLa cell lines were transfected with the indicated siRNA oligonucleotides and seeded in 8 well µ-slide chambers (Ibidi). The cells were imaged at the indicated times after transfection as follows: for Figure 1A,C, an LSM 5 live confocal microscope (Zeiss) equipped with 488-nm and 561-nm laser lines, a Plan-Apochromat 10× NA 0.45 objective and environmental control system (Ibidi, Gräfelfing, Germany) were used. ZEN software (Zeiss, Jena, Germany) was used to acquire images from five 7.5-µm-spaced optical Z-sections at various XY positions every three minutes. Single position *.tiff files were generated from the maximum intensity projections in ZEN and were converted to image sequences in Fiji. For Figure S2B, a tLSM5 live microscope with a Plan-Apochromat 20× NA 0.8 objective was used, acquiring images from five to eleven optical Z-sections at various XY positions every three minutes. Time-dependent quantitative measurements of VPS72 and IBB eGFP-fusions in the nuclear compartment were performed using Icy [37]. For Figure 1E,F, Figure 5A, Figure 7E, Figures S1, S9A and S9C used a Ti2 Eclipse (Nikon) equipped with a LED light engine SpectraX(Lumecor, Beaverton, OR, USA) and GFP/mCherry filter sets, a Plan-Apochromat 10× NA 0.5 objective and environmental control system (Ibidi). Elements software (Nikon) was used to perform fluorescence multi-position imaging every three minutes and the subsequent conversion to image sequences.

The analysis of dwell time in the different phases of mitotic progression was performed using CecogAnalyzer 1.5.2 (http://www.cellcognition.org/software/cecoganalyzer) as in [13,28]. Shortly, an expert biologist performed annotations based on the global differential morphologies of the chromatin throughout the cell cycle. Here, chromatin masses were annotated as telophase when they followed anaphase events timely and were clearly distinct from the anaphase and interphase morphologies of surrounding cells.

### 2.8. Sequence Alignment, Identity, Similarity Scores and Protein Blast

Human (NP_005988.1), mouse (NP_033362.2), *Xenopus* (NP_001085907.1), fruit fly (NP_001285823.1) and budding yeast (AHY75432.1) proteins were retrieved from the National Center for Biotechnology Information (NCBI) database. YL1-C domains (pfam08265) were also retrieved according to the NCBI annotations. Sequences were aligned by ClustalO with the Seaview software [38]. The alignment was used to calculate the identity and similarity scores of pairwise comparison for the full-length protein or the YL1-C domain alone. The calculations were performed through the sequence identity and similarity (SIAS) webpage of the computational University of Madrid, using the default settings.

Table S1 is the output of Standard Protein BLAST [39] of the Human YL-1 domain protein sequence (VCPVTHRPALYRDPVTDIPYATARAFKII; NP_001258016.1 (aa302-330) against the reference sequence protein (refsep_protein) databank restricted to Metazoa (taxid:33208). For additional protein Blast parameters, see Table S2 (YL-1 domain Protein Blast_A057CP8Z01R_search_strategy).

## 3. Results

### 3.1. VPS72 Is Required for Timely Mitotic Exit

We have previously identified two ATPases, pontin and reptin, also known as RuvBL1 and RuvBL2, as chromatin de-condensation factors with crucial functions during mitotic exit [21]. Consistent with data from cell free assays and the proposed function in chromatin de-condensation during mitotic exit, siRNA-mediated downregulation of either pontin, reptin or both in HeLa cells stably expressing H2B-mCherry and IBB-EGFP (the importin β binding domain of importin α, used as nuclear import substrate [40]) extended the time from anaphase onset to the end of telophase (Figure 1A,B and Figure S1A) as analyzed by life cell imaging and with the CellCognition software [28,41,42]. Especially telophase, based on the morphological annotation of chromatin, was extended upon pontin and/or reptin downregulation to 30, 39 and 36 min as compared to the control cells (21 min). However, pontin and reptin downregulation did not affect the timing of nuclear envelope reassembly after mitosis as measured based on the nuclear import substrate IBB-EGFP (Figure S1B). Because pontin and reptin interact with each other and can assemble hetero-dodecamers [43], the expression and/or stability of both proteins is interdependent and because of the essential nature of these proteins, their downregulation is only partial, as previously observed [44,45].

In a search for pontin/reptin cofactors required for mitotic exit, we transfected HeLa cells stably expressing H2B-mCherry and IBB-EGFP with siRNA oligos targeting several known pontin/reptin interactors with chromatin functions and performed a similar analysis (Figure 1C and Figure S1C). Downregulation of VPS72 and ASH2L4 significantly (*p* < 0.001) extended the time from anaphase onset to the end of telophase but did not strongly delay nuclear envelope reassembly (Figure S1D). When extending the analysis with three more siRNA oligos per candidate in HeLa cells stably expressing H2B-mCherry, the delay of mitotic exit could only be confirmed for VPS72 (Figure 1D–F and Figure S1E; downregulation of VPS72 was assayed with an antibody directed against the human protein). VPS72 is part of the pontin/reptin-containing SRCAP and EP400 chromatin remodeling complexes and acts as a H2A.Z chaperone or reader. Interestingly, downregulation of VPS72 especially extended telophase, while PP2A, as reported [13], delays the end of anaphase and extends telophase (Figure 1F).

### 3.2. VPS72 Accumulates on Chromatin during Mitotic Exit

**Figure 2:**
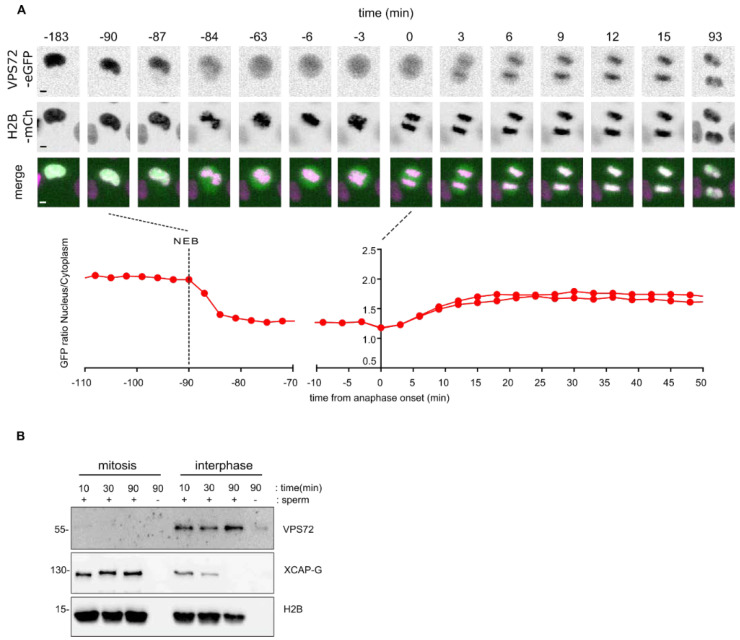
VPS72 accumulates on chromatin during mitotic exit: (**A**) Live cell imaging of mitotic progression of a HeLa cell stably expressing VPS72-EGFP and H2B-mCherry. Time is normalized to anaphase onset. The lower panel shows time-dependent nuclear-to-cytoplasmic ratio of the VPS72-EGFP signal as measured for the mitotic event shown above. NEB, nuclear envelope breakdown. Scale bars, 5 µm. (**B**) *Xenopus* VPS72 binds to chromatin in interphase. Mitotic (cytostatic factor arrested) or interphase *Xenopus* egg extracts were incubated where indicated with sperm chromatin for indicated time. The samples were diluted, and chromatin was re-isolated by centrifugation and analyzed by Western blotting.

To examine the localization of VPS72, we established HeLa cell lines stably co-expressing EGFP-tagged VPS72 and H2B-mCherry. Live cell imaging showed that VPS72, independent of whether the EGFP tag was *N*- or *C*-terminal, localizes in the nucleus during interphase (Figure 2A and Figure S2A), consistent with the reported nuclear localization of the protein [46]. Upon nuclear envelope breakdown, EGFP-tagged VPS72 distributes within the mitotic cytoplasm and weakly localizes to the condensed chromosomes. The chromatin localization increased during chromosome segregation in anaphase and chromatin de-condensation in telophase (Figure 2A and Figure S2B), consistent with the notion that VPS72 functions late in mitosis. VPS72 appears at the same time as the IBB-eGFP fusion on the chromatin (6–9 min after metaphase to anaphase transition) but with different accumulation kinetics (Figure 2A and Figure S2B).

To characterize the molecular function of VPS72 in *Xenopus* egg extracts, a versatile tool to biochemically characterize mitotic processes, we generated rabbit polyclonal antibodies against *Xenopus* VPS72 (see the Material and Methods section for details). Incubation of sperm chromatin in egg extracts and chromatin re-isolation showed that *Xenopus* VPS72 bound to chromatin in interphase but not in mitosis (Figure 2B). CAP-G, a component of the condensin complex, behaved expectedly the opposite way [32].

### 3.3. VPS72 Is Required for Nuclear Assembly and Organization

**Figure 3:**
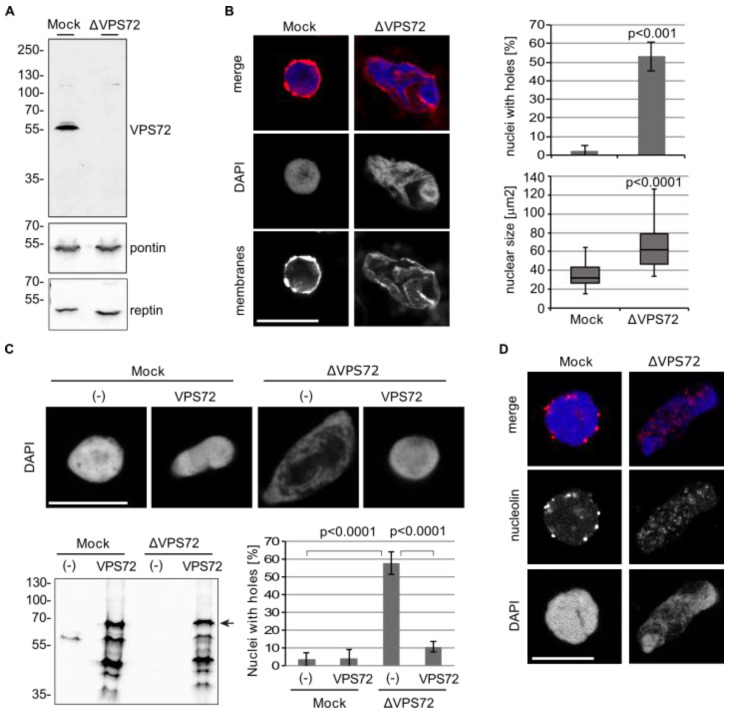
VPS72 is required for proper nuclear assembly and chromatin organization (**A**) Immunodepletion of VPS72 from *Xenopus* egg extracts. Control (Mock) and VPS72-depleted (ΔVPS72) extracts were immunoblotted for VPS72, pontin and reptin using *Xenopus* specific antibodies. (**B**) Confocal microscopy of nuclei assembled from sperm chromatin for 120 min in mock and VPS72-depleted *Xenopus* egg extracts. Membranes were pre-labelled with DiIC18 (1,1’-Dioctadecyl-3,3,3’,3’-Tetramethylindocarbocyanine Perchlorate, red in overlay). And chromatin was stained with DAPI (4,6-Diamidin-2-phenylindol, blue in overlay). Samples were fixed in 4% paraformaldehyde and 0.5% glutaraldehyde to optimally preserve chromatin structure. A confocal section of the center of representative nuclei is shown. Quantitation shows the percentage of nuclei with “holes”, i.e., containing areas devoid of DAPI staining (*n* = 3 experiments, *n* > 50 structures per experiment and condition) and the cross-sectional area (*n* = 3 experiments, *n* > 10 structures per experiment and condition). (**C**) Confocal microscopy images of nuclei assembled from sperm chromatin for 120 min in mock and VPS72-depleted egg extract, supplemented with buffer (−) or recombinant SUMO-tagged VPS72. Samples were processed and analyzed as in **B**. *n* = 3 experiments, *n* > 45 structures. The corresponding western blot shows mock and VPS72-depleted extracts supplemented with buffer or SUMO-tagged VPS72, the position of which is indicated by an arrow. (**D**) Nuclei were in vitro assembled on sperm chromatin with unlabeled membranes for 120 min and fixed in 4% paraformaldehyde. The assembled nuclei were stained with a *Xenopus* nucleolin antibody and DAPI. Scale bars, 10 µm. Error bars represent SD. Statistical significance was determined using two-tailed student’s test.

To gain insight into the function of VPS72 during nuclear assembly, we depleted the protein from *Xenopus* egg extracts. In this system, formation of a nucleus including an intact nuclear envelope and nuclear pore complexes can be faithfully reconstituted [22]. While endogenous VPS72 was efficiently depleted, the antibody-bead treatment did not affect the amount of reported VPS72-interacting proteins, such as pontin and reptin (Figure 3A). In both control (Mock) and VPS72-depleted extracts, a nuclear envelope formed around the chromatin (Figure 3B) including nuclear pore complexes and the lamina (Figure S3A). However, the chromatin within the nuclei was assembled in VPS72-depleted extracts with irregular shape; DAPI staining was heterogeneous with seemingly large unstained regions within the nucleus (observed as “holes” in 50% of the chromatin substrates, Figure 3B,C). In addition, membranes assembled not only on the chromatin surface but also inside the nucleus (Figure 3B and Figure S3A). The nuclei were also significantly larger than in control reactions (Figure 3B,C). Despite the irregular membrane appearance, the nuclei assembled in VPS72-depleted extracts contained a closed nuclear envelope: the nuclei excluded fluorescently labeled 70 kD dextrans. indicating an intact nuclear envelope [47], whereas 10 kDa dextrans, which can diffuse through nuclear pore complexes, entered the chromatin area (Figure S3B). Similarly, nuclear pore complex staining was normal when VPS72 was downregulated in HeLa cells (Figure S4F), consistent with a proper although delayed nuclear import function (Figure S1D). Indeed, also in vitro, in the absence of VPS72, assembled nuclei showed normal protein import (Figure S3C).

In the in vitro experiments, the nuclear structure defects were rescued by addback of recombinant VPS72, purified as a SUMO-tagged protein to increase solubility in bacteria, proving that the lack of VPS72 causes the defects (Figure 3D). Both, the apparently disorganized chromatin structure and the irregular membrane staining is lost in the rescue experiments.

To examine if nuclear morphology was impaired upon VPS72 depletion, we stained the nuclei for marker proteins. In control nuclei, nucleolar marker KI-67 was enriched at the rim of the nucleolus as expected (Figure S3A) [48]. Upon VPS72 depletion, the circle-like KI-67 staining became much bigger and intense, indicating a problem in nucleolar assembly [49].

Nucleoli markers such as nucleolin are in this assay localized as a small number of dots on the chromatin [50]. Upon VPS72 depletion, the staining was dispersed and scattered all over the chromatin area (Figure 3D), indicating that VPS72 is required for proper nuclear organization. In HeLa cells, downregulation of VPS72 did not significantly change KI-67 staining during mitotic exit (Figure S5). In interphase, the amount of KI-67 in the nucleus, the number of nucleoli per nucleus and the size of the nucleoli were also unaffected by VPS72 downregulation (Figure S4E). In line with this, nucleolar integrity and nucleolar RNA synthesis were not affected by VPS72 downregulation (Figure S6).

Histone marks for euchromatin (H3K4me3) and heterochromatin (H3K9me3) as well as HP1α (Figure S3A) were present in the absence of VPS72-assembled nuclei without significant alterations. Consistently, staining of chromatin markers remained unchanged in HeLa cells upon VPS72 downregulation (Figure S4A–D).

We questioned if the abnormal nuclei assembled upon VPS72 depletion are functionally intact. To test this, we performed the nuclear assembly reaction in the presence of Cy3-labeled dUTP to monitor DNA replication as one of the main nuclear functions. While the control extract incorporates dUTP on chromatin, the VPS72-depleted extract did not, indicating that DNA replication does not occur in these abnormal nuclei (Figure S7).

**Figure 4:**
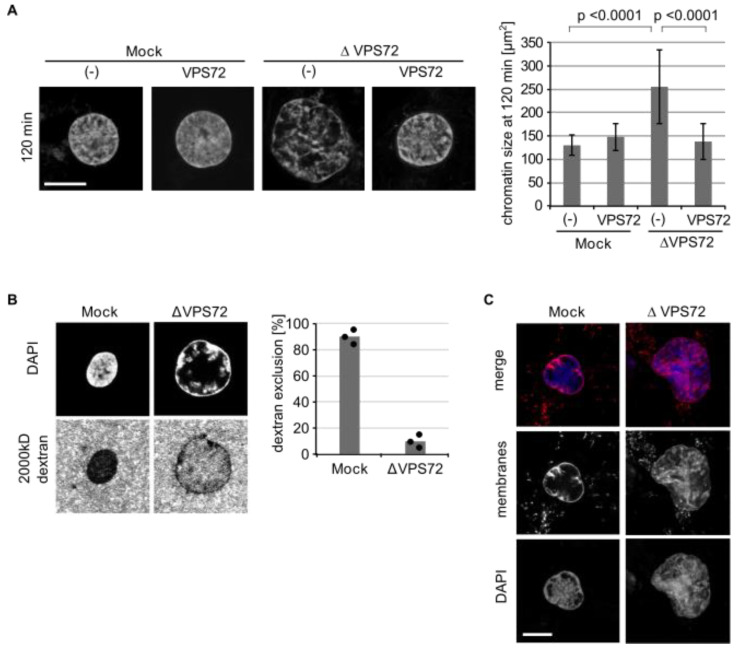
VPS72 is required to assemble compact nuclear chromatin structures: (**A**) Mitotic chromatin clusters from HeLa cells were incubated with mock or VPS72-depleted *Xenopus* egg extracts supplemented with buffer or recombinant (SUMO-tagged) VPS72 in the absence of membranes. After 120 min, samples were fixed with 4% paraformaldehyde and 0.5% glutaraldehyde, stained with DAPI and analyzed by confocal microscopy. The cross-sectional area of the chromatin substrates was quantified using *n* = 3 experiments, *n* > 10 structures per experiment and condition. (**B**) Mitotic chromatin clusters from HeLa cells were incubated with mock or VPS72-depleted *Xenopus* egg extracts for 120 min. At the end of the reaction, fluorescently labeled 2000 kDa dextran and DAPI were added without fixation and samples were immediately analyzed by confocal microscopy. Quantitation shows the average percentage of dextran chromatin structures from three independent experiments, each counting 20 chromatin substrates per sample and condition. Individual data points are indicated. (**C**) Mitotic chromatin clusters from HeLa cells were incubated in mock or VPS72-depleted egg extracts in the presence of DiIC18-labelled membranes (red in overlay). Samples were fixed with 4% paraformaldehyde and 0.5% glutaraldehyde after 120 min, stained with DAPI (blue in overlay) and analyzed by confocal microscopy. Scale bars, 10 µm. Error bars represent SD. Statistical significance was determined using two-tailed student’s test.

To confirm the defects in nuclear organization, we used mitotic chromatin clusters as an alternative chromatin template instead of sperm DNA. When mitotic chromatin clusters are incubated in egg extracts, the chromatin decondenses and assembles interphase chromatin structures, even in the absence of membranes [21]. Upon VPS72 depletion, the nuclei formed in this assay were significantly larger and showed a more heterogeneous DAPI labeling, an effect that can be rescued by the addition of recombinant VPS72 (Figure 4A). The larger chromatin structures upon VPS72 depletion appeared less compact in DAPI staining. Indeed, when a large size dextran was added to the reaction, it was not efficiently excluded from the chromatin upon VPS72 depletion in contrast to control reactions (Figure 4B). When membranes were added to the reaction, they formed a nuclear envelope around the chromatin as reported before [21]. However, upon VPS72 depletion, the membranes invaginated into the chromatin area (Figure 4C), consistent with the phenotype observed on sperm chromatin as a template (Figure 3). Similar to the situation with sperm chromatin, histone marks for euchromatin (H3K4me3) and heterochromatin (H3K9me3 and H3K27me2) as well as HP1α were present on in vitro decondensed chromatin clusters without significant alterations upon VPS72 depletion (Figure S8).

### 3.4. VPS72 Performs Its Function Outside of Remodeling Complexes

**Figure 5:**
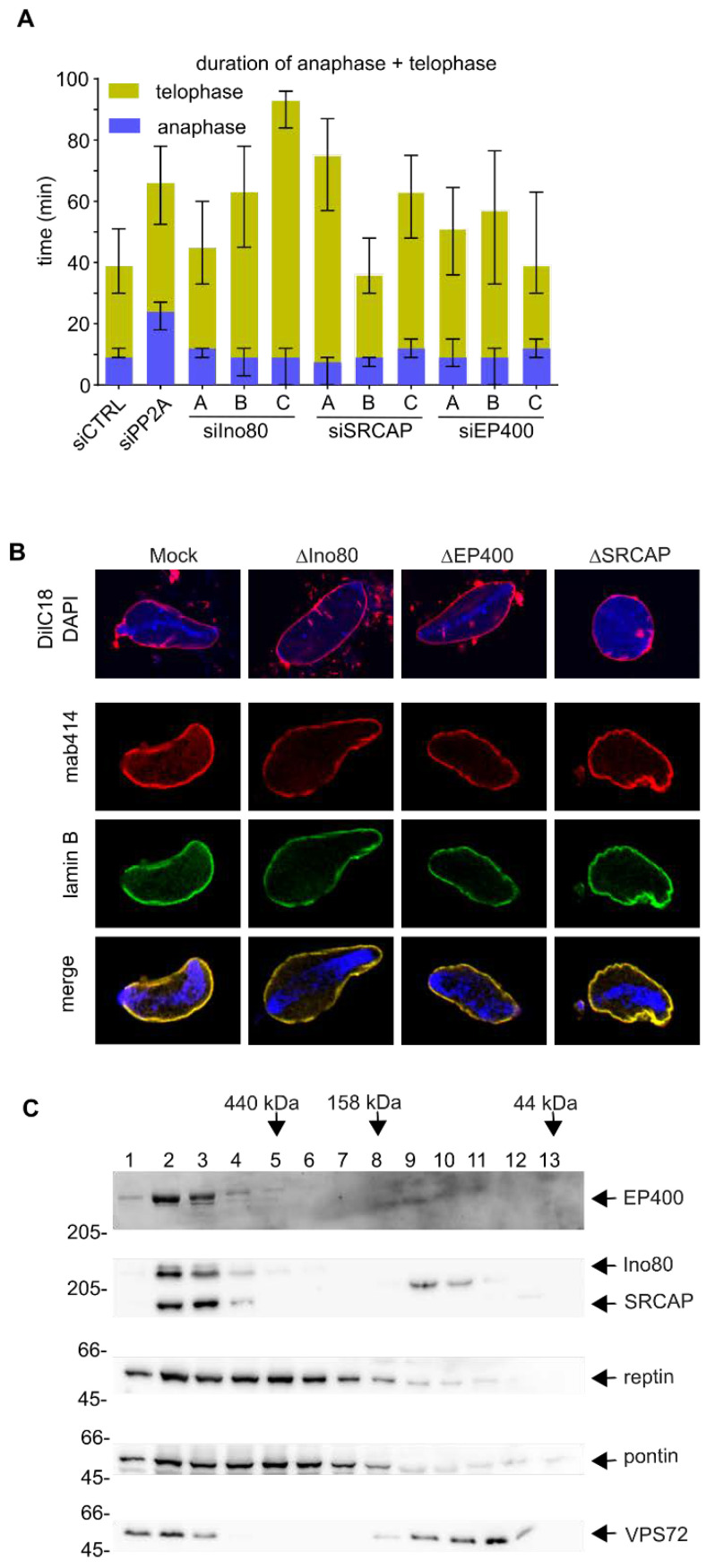
VPS72 performs its function in nuclear assembly outside of the SRCAP and EP400 chromatin remodeling complexes. (**A**) HeLa cells expressing mCherry-H2B were transfected with 20 nM siRNA oligos against SRCAP, Ino80 or EP400; 48−96 h after transfection, cells were analyzed by life cell imaging and the length of mitotic exit (time from metaphase to anaphase transition until end of telophase) was determined. The median time spent in late anaphase and telophase is shown for mitotic events from 2 independent experiments. Colored bars indicate medians, and error bars represent interquartile range. Statistical significance at alpha = 0.001 was determined using a Kruskal–Wallis test followed by Dunn’s multiple comparisons test. (**B**) Confocal microscopy of nuclei assembled from sperm chromatin for 120 min in mock *Xenopus* egg extracts or depleted for SRCAP, EP400 or Ino80: In the upper panel, membranes were pre-labelled with DiIC18 (red in overlay) and chromatin was stained with DAPI (blue in overlay). In the lower panel, nuclear pore complexes were labeled with mAB414 (red) and lamin B (green) and. in the merge. DAPI is shown in blue. (**C**) *Xenopus* egg extracts were separated on a Superdex 200 column, and fractions were analyzed with indicated antibodies. A substantial portion of VPS72 is found separated from the EP400 and SRCAP complexes. Molecular size markers of the calibration of the Superdex 200 column are indicated on the top.

VPS72 is reportedly part of two chromatin remodeling complexes, the SRCAP and EP400 complex, the latter also known as the TRRAP/TIP60 complex [51]. We wondered whether VPS72 needs to be part of these larger complexes in order to fulfill its function in nuclear reformation. We downregulated the expression of the main ATPases of SRCAP and EP400 (SRCAP or p400/hDomino), which distinguish these complexes, in HeLa cells (Figure 5A). As a control, we included Ino80, an ATPase of unrelated chromatin remodeling complexes, which does contain pontin and reptin but not VPS72. Downregulation of these three ATPases, EP400, SRCAP and Ino80, resulted in telophase extension (Figure 5A) similar to that observed upon VPS72 downregulation, but the different oligos affected the cells to different degrees despite similar downregulation efficacy as judged by western blotting (Figure S9B). Importantly, downregulation of the three ATPases impaired cell viability (Figure S9A) consistent with their crucial multilevel function in cell homeostasis [27], which complicates interpretation of the analysis.

We therefore used the *Xenopus* egg extract system, which, as a cell free system, is not affected by cell viability, to analyze these ATPases in nuclear reassembly. All three ATPases could be efficiently depleted from egg extracts without significantly affecting VPS72 levels (Figure S9D). In all depletion conditions, nuclei undistinguishable from the mock control formed and did not show chromatin structure deviations observed upon VPS72 depletion (Figure 5B). This indicates that VPS72 acts during nuclear reformation outside of these chromatin remodeling complexes, at least in cell free assays. Indeed, size exclusion chromatography shows that a large fraction of VPS72 in egg extracts does not co-elute with SRCAP or EP400 (Figure 5C).

### 3.5. VPS72-Mediated H2A.Z Chromatin Loading Is Required for Nuclear Assembly

**Figure 6:**
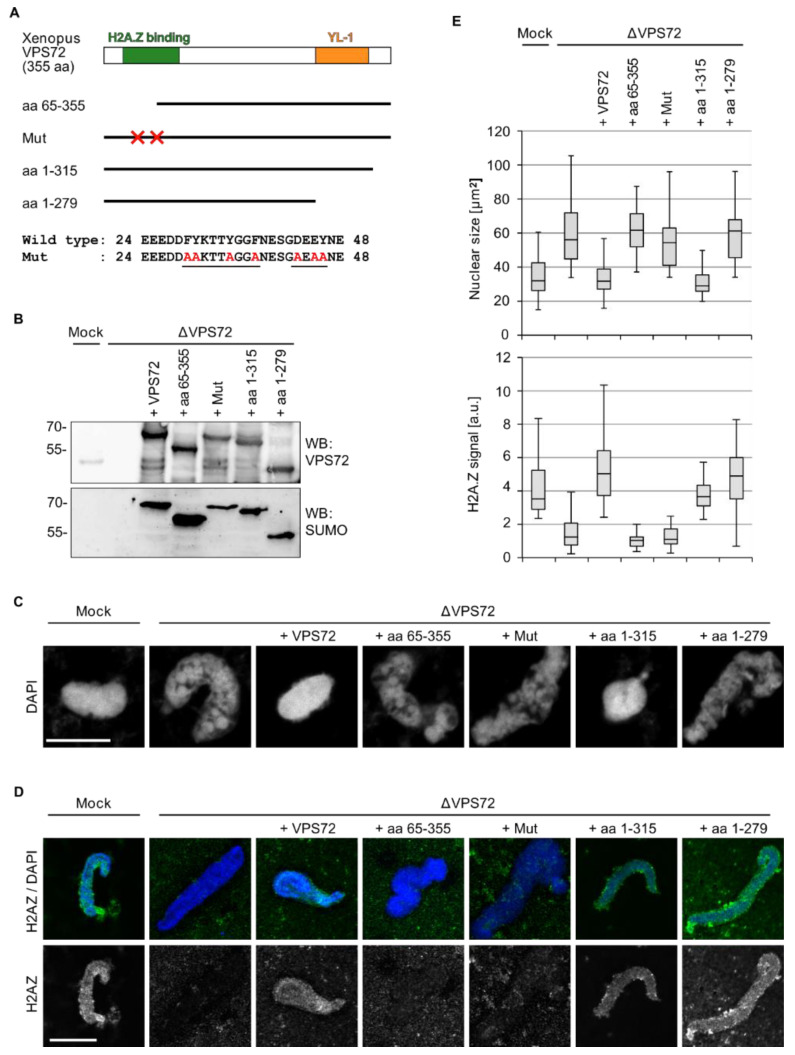
H2A.Z-binding and the YL-1 domain are required for VPS72 function in nuclear assembly. (**A**) Domain structure of VPS72, including deletion fragments and a mutant abolishing the H2A.Z interaction employed: The amino acid sequence changes in this mutant are indicated below. (**B**) VPS72-depleted extracts were supplemented with comparable amounts of recombinant SUMO-tagged proteins, as indicated. Samples were analyzed by western blotting with VPS72 and SUMO antibodies. (**C**) Confocal microscopy images of nuclei assembled from sperm chromatin for 120 min in mock and VPS72-depleted *Xenopus* egg extracts supplemented with buffer or different VPS72 constructs: Samples were fixed with 4% paraformaldehyde and 0.5% glutaraldehyde and stained with DAPI. Scale bars, 10 µm. (**D**) H2A.Z staining of nuclei assembled from sperm chromatin for 120 min in mock and VPS72-depleted *Xenopus* egg extracts supplemented with buffer or different VPS72 constructs: Samples were fixed with 4% paraformaldehyde, were processed for immunostaining with H2A.Z antibodies and, after DAPI labeling, were analyzed by confocal microscopy. Scale bars, 10 µm. (**E**) Quantitation of the chromatin cross-sectional area from experiments done as in (**B**) and of the H2A.Z intensity from experiments from experiments as in C: *n* = 2 experiments, *n* > 10 structures per experiment and condition. For nuclear size, the difference between ΔVPS72 and mock, +VPS72 or +aa 1-315 have significance values *p* < 0.0001 and, between ΔVPS72 and +aa 63–355, +Mut or +aa 1–279, differences are not significant, tested by two tailed student’s test. For H2A.Z staining intensity, the difference between ΔVPS72 and mock, +VPS72, +aa 1–315 or +aa 1–279 have significance values *p* < 0.0001 and, between ΔVPS72 and +aa 63-355 or +aa 1–279, differences are not significant, tested by two tailed student’s test.

To assess which regions within VPS72 are required for its function in nuclear assembly, we tested different VPS72 truncations (Figure 6A) in the *Xenopus* egg extract system. When full-length VPS72 was added, the depletion phenotype was rescued as before, leading to normal sized and organized chromatin structures (Figure 6B,C,E). In contrast, a VPS72 fragment lacking the *N*-terminal 64 aa was not able to substitute endogenous VPS72. The *N*-terminal domain of VPS72 comprises the H2A.Z binding domain [29,30], suggesting that H2A.Z loading is involved in VPS72 action in nuclear assembly despite the fact that SRCAP is not required for this function (Figure 5B). Indeed, a VPS72 mutant defective in H2A.Z binding, which was created based on the H2A.Z-VPS72 co-structure [30], was similarly unable to replace the endogenous protein in nuclear assembly. Both the *N*-terminal truncation and the mutant did not allow for H2A.Z integration into the chromatin substrate (Figure 6D).

**Figure 7:**
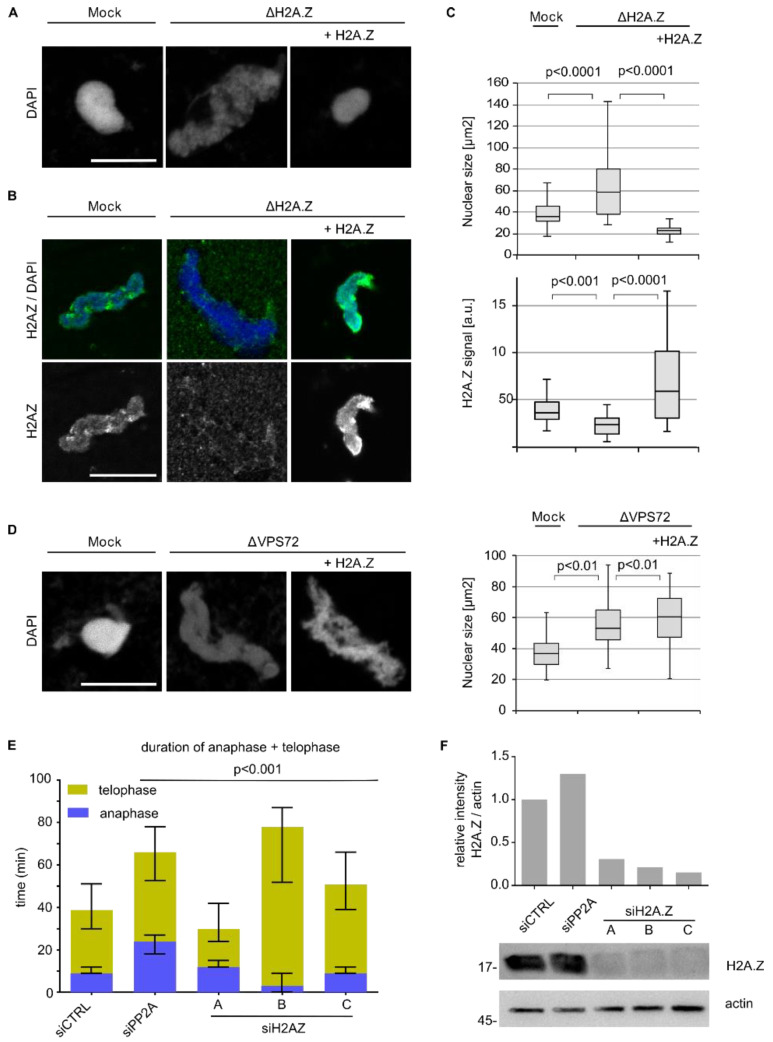
H2A.Z depletion mimics the nuclear assembly defects observed in VPS72 depletion. (**A**) Egg extracts were treated with either control or VPS72 aa 1-97 beads to deplete H2A.Z (ΔH2A.Z) and were supplemented where indicated with recombinant H2A.Z-H2B. The extracts were incubated with sperm chromatin for 120 min, fixed with 4% paraformaldehyde and 0.5% glutaraldehyde, stained with DAPI and analyzed by confocal microscopy. Scale bars, 10 µm. (**B**) Nuclei were assembled as in A, were fixed in 4% paraformaldehyde, were stained for H2A.Z and DAPI, and were analyzed by confocal microscopy. Scale bar, 10 µm. (**C**) Nuclear size and H2A.Z signal were quantified as in Figure 6E. *n* = 3 experiments, *n* > 20 structures per experiment and condition. Statistical significance was determined using two-tailed student’s test. (**D**) VPS72 was depleted from egg extracts and supplemented, where indicated, with 1 µM recombinant H2A.Z-H2B. Extracts were incubated with sperm chromatin for 120 min, were fixed with 4% paraformaldehyde and 0.5% glutaraldehyde, were stained with DAPI and were analyzed by confocal microscopy. Nuclear size was quantified as in C. (**E**) HeLa cells expressing mCherry-H2B were transfected with 20 nM siRNA oligos against H2A.Z; 24–72 h after transfection, cells were analyzed by life cell imaging and the length of mitotic exit (time from metaphase to anaphase transition until end of telophase) was determined. Colored bars indicate medians and error bars represent interquartile range. Statistical significance at alpha = 0.001 was determined using a Kruskal–Wallis test followed by Dunn’s multiple comparisons test. (**F**) Western blot and quantitation show the downregulation of H2A.Z at 48 h post-transfection (siRNA 20 nM) in HeLa cells stably expressing mCherry-H2B.

These data suggested that H2A.Z chromatin integration is crucial for VPS72 function in nuclear assembly. To test this hypothesis, we directly depleted H2A.Z from egg extracts using the H2A.Z binding *N*-terminal VPS72 domain. Indeed, depletion of H2A.Z resulted in an identical phenotype as compared to VPS72 depletion; the nuclei assembled in vitro were larger, showed a disorganized chromatin organization (Figure 7A,C) and were expectedly devoid of an H2A.Z signal (Figure 7B,C). Similar, to VPS72 depletion, H2A.Z-depleted nuclei were not able to replicate their DNA (Figure S7A). H2A.Z depletion did not cause a co-depletion of VPS72 (Figure S7B). Indeed, re-addition of the purified recombinant H2A.Z–H2B complex rescued the depletion phenotype, showing the specificity of the H2A.Z depletion. Nevertheless, even an excess of the H2A.Z–H2B complex added to VPS72 depleted extracts could not bypass the need for VPS72 in assembling a proper nuclear chromatin structure (Figure 7D).

RNAi-mediated H2A.Z downregulation in cells stably expressing H2B-mCherry showed different effects on telophase extension depending on the siRNA oligo used (Figure 7E,F). However, these siRNA oligos also affected cell viability differently, so that off-target effects cannot be excluded (Figure S9A), which complicates interpretation of the analysis. However, H2A.Z downregulation with oligo C extends telophase (10 min in median time over control) while having no impact on HeLa cell viability (Figure 7E,F and Figure S9A), which is a phenotype reminiscent of VPS72 downregulation. Similar to VPS72 downregulation, H2A.Z downregulation does not seem to impact barrier to autointegration factor 1 (BAF1) recruitment on chromatin during mitotic exit (Figure S10B).

### 3.6. The Conserved YL-1 Domain Is Required for VPS72 Function in Nuclear Assembly

Whereas an N-terminal truncation of VPS72 is nonfunctional in nuclear assembly because of the requirement for H2A.Z chromatin incorporation, a *C*-terminal VPS72 truncation lacking 40 aa rescued the nuclear assembly phenotype (Figure 6C,D). However, further *C*-terminal truncations deleting the conserved YL-1 domain, which is also referred to as YL-1-C domain, yield a protein that could not rescue the chromatin structure phenotype, despite the fact that H2A.Z was still integrated in the chromatin template (Figure 6C). This might indicate that the YL-1 domain of VPS72 has a crucial function in nuclear reassembly. Interestingly, within VPS72, the YL-1 domain shows a relative high sequence conservation (Figure S11). This domain shows 41% sequence identity and 55% sequence similarity between humans and budding yeast, whereas the entire protein possesses only 21% sequence identity and 31% sequence similarity between both species. In addition, within the more similar metazoan VPS72 proteins (e.g., 36% sequence identity between humans and drosophila), the YL-1 domain is a region of higher sequence conservation (52% sequence identity between humans and drosophila). This would be congruent with an important and conserved function of the YL-1 domain, which in metazoans, to date, is known to be present only in VPS72 proteins. What this function is remains an interesting path for future research.

## 4. Discussion

Here, we show that VPS72 is required for nuclear reassembly at the end of mitosis via its action on H2A.Z. Downregulation of VPS72 or H2A.Z extends telophase in HeLa cells. More profoundly, in *Xenopus* egg extracts, the depletion of VPS72 or H2A.Z results in nuclei with malformed nuclear envelope and disorganized chromatin. Importantly, the addback experiments using recombinant VPS72 or H2A.Z-H2B complex reverting VPS72 or H2A.Z depletion demonstrate that the depletion phenotype is due to the specific lack of either VPS72 or H2A.Z, respectively.

Intriguingly, the striking phenotypes observed in the cell free system do not equal strong phenotypes in living cells beyond the significantly extended telophase. Cells where VPS72 is downregulated, even by RNAi treatment for three days, do not show obvious chromatin phenotypes, defective nuclear envelope recruitment (Figures S4 and S10), nucleolar function defects (Figure S5), differences in nucleolar or chromatin state markers (Figures S4 and S5) or affected cell viability (Figure S9A). This lack of penetrance in living cells could be explained by a lower efficiency of VPS72 downregulation compared to the depletion in the in vitro experiments. Alternatively, the role of VPS72 in nuclear reassembly during mitotic exit in human cells might be, at least in part, redundant.

Human VPS72, also referred to as YL1, YL-1 or Swc2, was initially identified as a nuclear, DNA-binding protein that suppressed anchorage-independent growth suppressor activity in Kirsten sarcoma virus-transformed NIH3T3 cells [46,52]. In budding yeast, the VPS72 orthologue Swc2 was identified as part of the SWR1 chromatin remodeling complex and was implicated in the ATP-dependent replacement of H2A by H2A.Z [53,54,55]. In Drosophila, VPS72 is found in the acetyl-transferase complex TIP60 [56], whereas in mammals, it is shared by two chromatin-remodeling complexes: the EP400 complex, the mammalian TIP60 equivalent, and the SRCAP complex, the mammalian SWR1 correspondent [51]. VPS72 is essential for binding and transferring H2A.Z in both the yeast SWR1 [57,58] and the human SRCAP [29,30] complexes.

The observed function of VPS72 in nuclear reformation we report here is indeed linked to H2A.Z. A VPS72 truncation lacking the H2A.Z binding site as well as a mutant defective in the H2A.Z binding cannot substitute the wild-type protein in *Xenopus* egg extracts (Figure 6). Importantly, H2A.Z depletion copies the phenotype observed upon VPS72 depletion in cell free assays (Figure 7). However, VPS72 and H2A.Z function in nuclear reassembly does not require the SRCAP complex itself. Depletion of the SRCAP ATPase does not impact nuclear reassembly indicating that, at least in this system, VPS72 can perform its function in nuclear reassembly outside of this chromatin remodeling complex (Figure 5). This notion is supported by the fact that depletion of pontin and reptin, which are part of both the SRCAP and EP400 remodeling complex, among many other cellular machineries, do not lead to the same phenotype as VPS72 depletion. We have previously shown that depletion of pontin and reptin blocks chromatin de-condensation using mitotic chromosomes in egg extracts [21]. When instead sperm heads were used as chromatin template, the nuclei were undistinguishable from the control reactions [21]. Thus, VPS72 function in nuclear reassembly is similarly independent of pontin and reptin despite the fact that we originally identified VPS72 extending telophase in HeLa cells similar to pontin and reptin (Figure 1).

H2A.Z is an highly conserved histone variant [59] that has been implicated in heterochromatin regulation [60,61,62], anti-silencing function at heterochromatin boundaries in yeast [63], DNA repair [64], chromatin segregation [61] and transcriptional regulation [65,66,67]. This histone variant is loaded to replace the canonical H2A in nucleosomes at specific regulatory elements of the genome in a cell-cycle dependent manner [68], where it influences local chromatin structure [69]. We speculate that loss of this structural organization brought about by H2A.Z impacts nuclear reassembly and causes the defects seen upon VPS72 and/or H2A.Z depletion. Vertebrates possess two H2A.Z encoding genes (H2AFZ and H2AFV) which give rise to two proteins, H2A.Z.1 and H2A.Z.2, that differ by three amino acids [70,71]. Depletion from *Xenopus* egg extracts performed with the H2A.Z binding domain of VPS72 removes, in all likelihood, both proteins. In HeLa cells, downregulation of H2A.Z.1 by siRNA extends the telophase, similar to VPS72 downregulation. The combined application of siRNA oligos against transcripts of both H2A.Z encoding genes did not further prolong telophase (data not shown).

In summary, we describe here an unexpected role of H2A.Z and its chaperone VPS72 in nuclear reformation. This function is independent of the SRCAP and EP400 chromatin remodeling complexes. Interestingly, the in vitro experiments suggest that H2A.Z deposition on chromatin is required but not sufficient for formation of a fully functional nucleus. This requires within VPS72 the YL-1 domain, which is a highly conserved part in VPS72 proteins. To assign the molecular function of this conserved domain remains an interesting task for future research.

## Figures and Tables

**Table 1:** siRNA oligos used in this work.

Name	Oligo Ref.	Target Sequence 5′-3 (Sense)	Manufacture Ref.	Manufacturer
**AllStars neg. control**			Cat. #1027281	Qiagen
**PPP2R1A_7**	triple	GACCAGGATGTGGACGTCAAA	SI04436495	Qiagen
**PPP2CA_5**	triple	ATGGAACTTGACGATACTCTA	SI02225783	Qiagen
**PPP2R2A_5**	triple	CTGCAGATGATTTGCGGATTA	SI02225825	Qiagen
**NUFIP1**		CCUAUGCUCACUAAUGAGUAGCUAU	HSS120217	Invitrogen
**YEATS4**		CCUGUAACCCUGUAUCAUUUGCUAA	HSS111971	Invitrogen
**HDAC3**		GCAACCCAGCUGAACAACAtt	120349	Invitrogen
**ASH2L**		GAUAAAUACUGGGAGUGCAUGACAA	HSS113411	Invitrogen
**VPS72**		GCCGAGUAGUCACCAAGGCCUAUAA	HSS110566	Invitrogen
**VPS72**	A	CAGCUGAGCAUACACGACAAACGUU	HSS186242	Invitrogen
**VPS72**	B	GAAGAUGAGUUCUACCAGATT	S13908	Invitrogen
**VPS72**	C	CCUUCAAGAUCAUUCGUGATT	S13909	Invitrogen
**hINO80**	A	GCAAGGGAAAUAAUGUUCCUGGGAA	HSS123095	Invitrogen
**hINO80**	B	CGACAAACGUCAGCUAUCUUCAAUA	HSS182590	Invitrogen
**hINO80**	C	CAGAAUAUGAAAGGCGAGUUCUGAA	HSS182591	Invitrogen
**SRCAP**	A	GCGUGAUGUUGAACUGGGAGAUGGA	HSS116728	Invitrogen
**SRCAP**	B	GCGCCUCAUUCUAUCUCCCGAUAUG	HSS116729	Invitrogen
**SRCAP**	C	CCCUCCUUCACAGAUUCCUCCUUGU	HSS116730	Invitrogen
**EP400**	A	CCAGUCUAUGGCAGAGACUUGCUAA	HSS126583	Invitrogen
**EP400**	B	GGGAGAUGCAAAGACAUCCACAUAU	HSS126584	Invitrogen
**EP400**	C	GGGCAAGGAGCAGAAGAAGAAUAUU	HSS126585	Invitrogen
**H2A.Z**	A	GCUAUUGAUUCUGAAGUAGUGGGUU	HSS142376	Invitrogen
**H2A.Z**	B	CCACUCUGGUGGAUAAGUUCAAUAA	HSS142377	Invitrogen
**H2A.Z**	C	UGGGCCGUAUUCAUCGACACCUAAA	HSS179165	Invitrogen

## Supplementary Materials

The following data are available online at https://www.mdpi.com/2073-4409/9/7/1702/s1, Figure S1: VPS72 functions in mitotic exit in HeLa cells, Figure S2: VPS72 accumulates on chromatin during mitotic exit; Figure S3: Depletion of VPS72 does not abolish nuclear and chromatin marks Figure S4: Downregulation of VPS72 does not affect chromatin markers in HeLa Cells, Figure S5: Downregulation of VPS72 does not affect KI-67 distribution during mitotic exit in HeLa cells, Figure S6: Downregulation of VPS72 does not affect nucleolar integrity and function in HeLa cells, Figure S7: VPS72 or H2A.Z depletion results in DNA replication defects, Figure S8: Depletion of VPS72 does not affect chromatin marks on in vitro decondensed chromatin, Figure S9: Downregulation of Ino80, SRCAP or EP400 compromises cell viability, Figure S10: Downregulation of VPS72 does not affect nuclear envelope recruitment during mitotic 168 exit in HeLa cells, Figure S11: Sequence alignment, identity and similarity scores of VPS72 full length and YL-1 179 domain, Table S1: Standard protein BLAST of the human YL-1 domain protein sequence, Table S2: YL-1 domain protein blast A057CP8Z01R search strategy.
