# Supplementary material for: VPS72/YL1-Mediated H2A.Z Deposition Is Required for Nuclear Reassembly after Mitosis
Source: Cells. 2020 Jul 16;9(7):1702. doi: 10.3390/cells9071702 (PMC7408173; doi:10.3390/cells9071702)
Supplement: Supplementary file 1 [file cells-09-01702-s001.zip › MorenoAndres_Supplement_15072020/VPS72_Supplemetary Figures_15072020.pdf]

cells MDPI

Supplementary Figures

VPS72/YL1-Mediated H2A.Z Deposition Is Required for Nuclear Reassembly after Mitosis

Daniel Moreno-Andrés [1,2,*,†], Hideki Yokoyama [1,2,†], Anja Scheufen [1], Guillaume Holzer [1], Hongqi Lue [1], Anna Katharina Schellhaus [1], Marion Weberruss [1,2], Masatoshi Takagi [3] and Wolfram Antonin[1,2,*]

[1] Institute of Biochemistry and Molecular Cell Biology, Medical School, RWTH Aachen University, 52074 Aachen, Germany; hideki-yokoyama@idpharma.jp (H.Y.); anscheufen@ukaachen.de (A.S.); gholzer@ukaachen.de (G.H.); hlue@ukaachen.de (H.L.); katharina.schellhaus@ki.se (A.K.S.); marion.weberruss@gmx.de (M.W.)

[2] Friedrich Miescher Laboratory of the Max Planck Society, Spemannstrasse 39, 72076 Tübingen, Germany

[3] Cellular Dynamics Laboratory, RIKEN Cluster for Pioneering Research, 2-1 Hirosowa, Wako, Saitama 351-0198, Japan; mtakagi@riken.jp

* Correspondence: dmoreno@ukaachen.de (D.M.-A.); wantonin@ukaachen.de (W.A.)

† These authors equally contributed to the work.

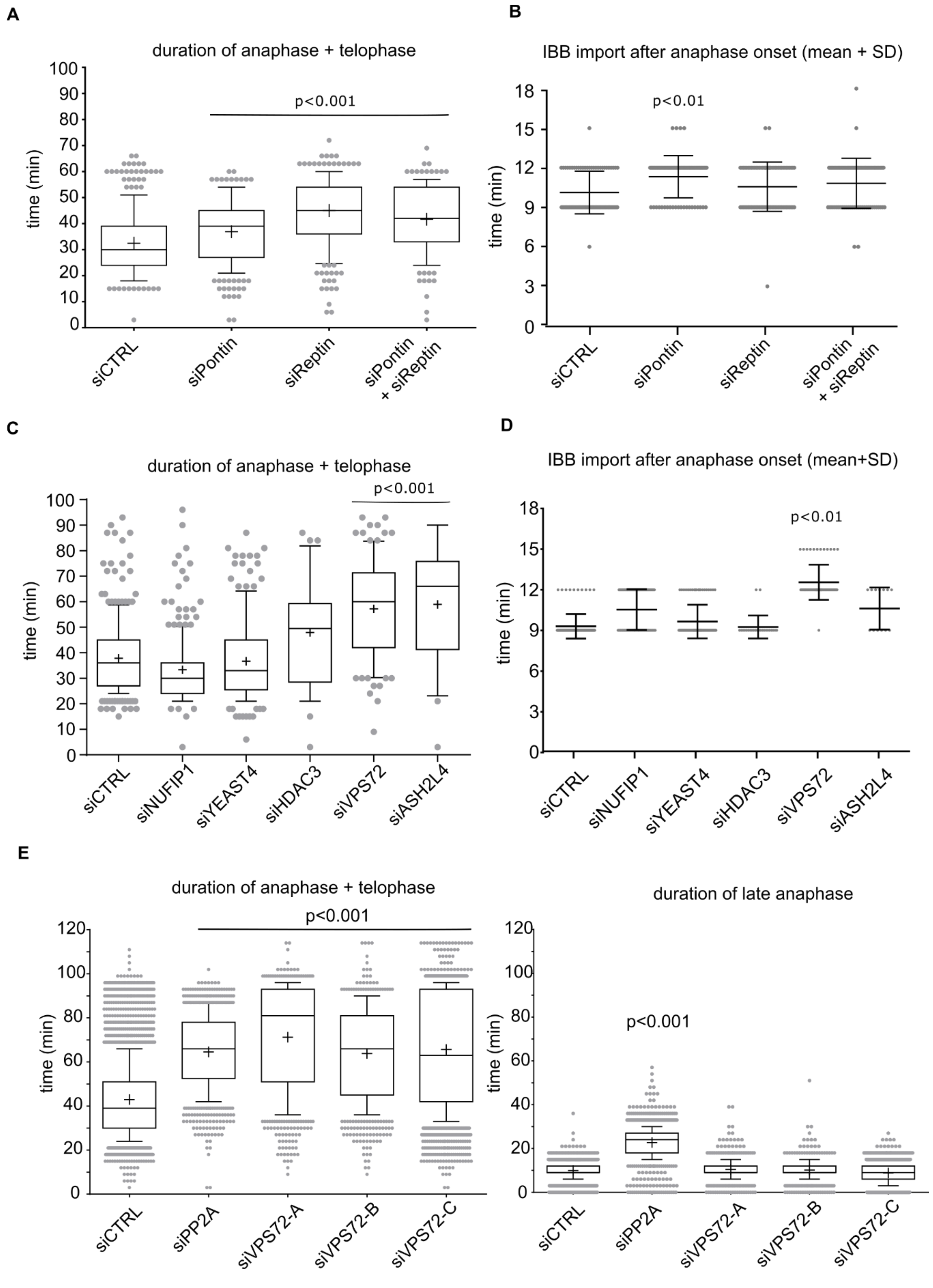

Figure S1. VPS72 functions in mitotic exit in HeLa cells. (a) Box blot presentation of the data presented in Figure 1A. **(b)** Onset of nuclear import, measured by nuclear accumulation of EGFP-IBB (importin β binding domain of importin α as a nuclear import substrate) in the cells analyzed in Figure 1A, after metaphase to anaphase transition. **(c)** Box blot presentation of the data presented in Figure 1C. **(d)** Onset of nuclear import, measured by nuclear accumulation of EGFP-IBB in the cells analyzed in Figure 1C, after metaphase to anaphase transition. **(e)** Box plot of the data presented in Figure 1F.

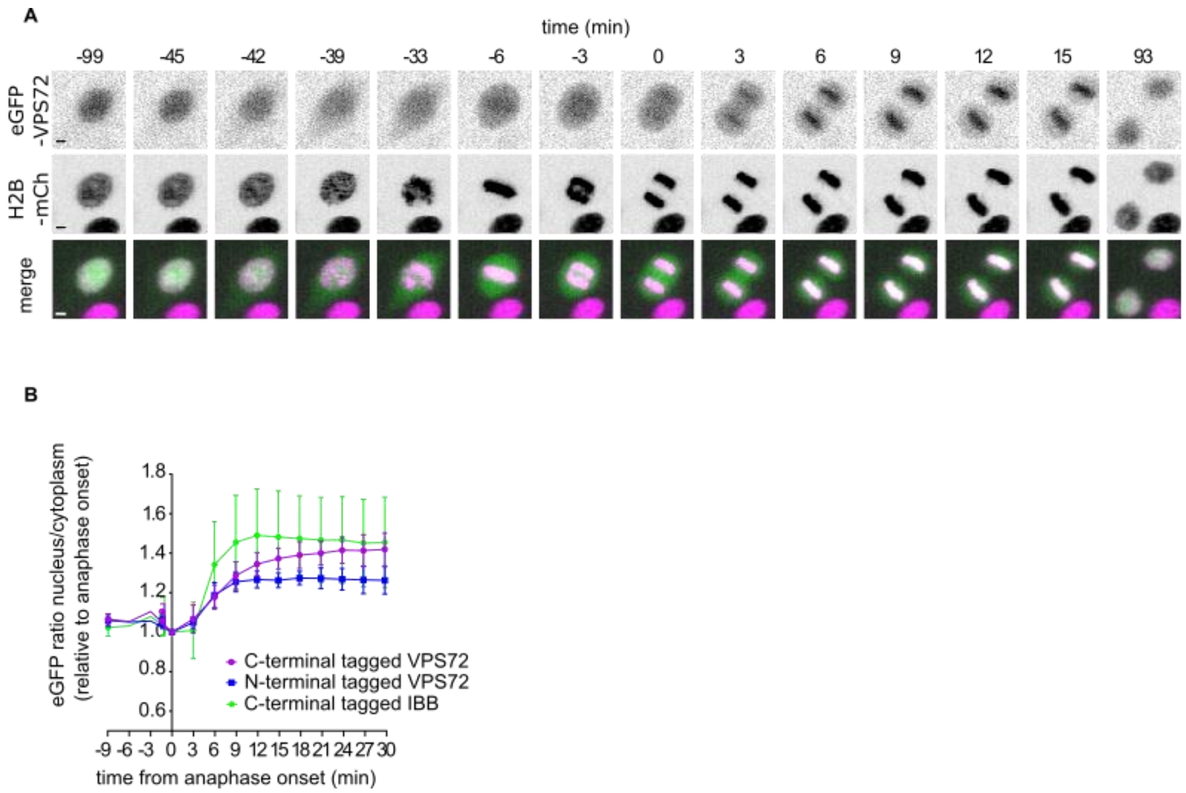

Figure S2. VPS72 accumulates on chromatin during mitotic exit. (a) Live cell imaging of a mitotic HeLa cell stably expressing EGFP-VPS72 and H2B-mCherry. Time is normalized to anaphase onset. **(b)** Nuclear to cytoplasmic ratio of the EGFP signal of HeLa cells stably expressing H2B-mCherry and N- or C-terminal EGFP-tagged VPS72 during passage though mitosis. Time and signal ratio of C-terminal tagged (magenta) and N-terminal tagged VPS72 (blue) and C-terminal tagged IBB (green) are normalized to anaphase onset in 6 cells per condition. Mean and SD is shown.

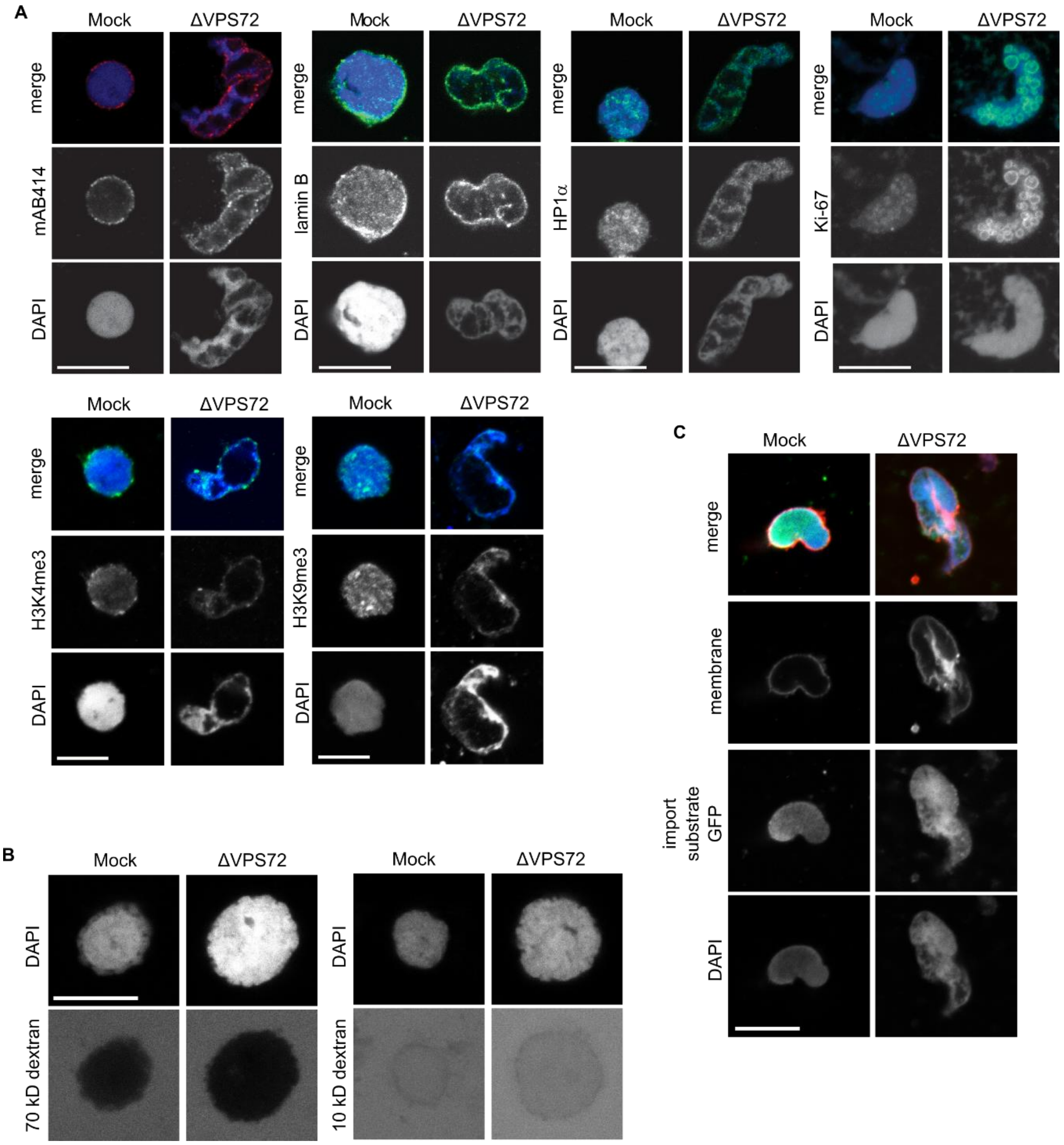

Figure S3. Depletion of VPS72 does not abolish nuclear and chromatin marks. (a) Nuclei were assembled as in Figure 3B, fixed with 4% paraformaldehyde and stained with indicated antibodies, chromatin was stained with DAPI, bar 10 µm. **(b)** Nuclei were assembled as in Figure 3B for 120 min. The samples were supplemented with 70 or 10 kD dextran and immediately imaged by confocal microscopy to check for nuclear envelope and nuclear pore complex integrity. **(c)** Nuclei were assembled as in Figure 3B for 90 min and an EGFP-tagged nuclear import substrate was added for 30 min. Reactions were stopped by fixation with 4% paraformaldehyde.

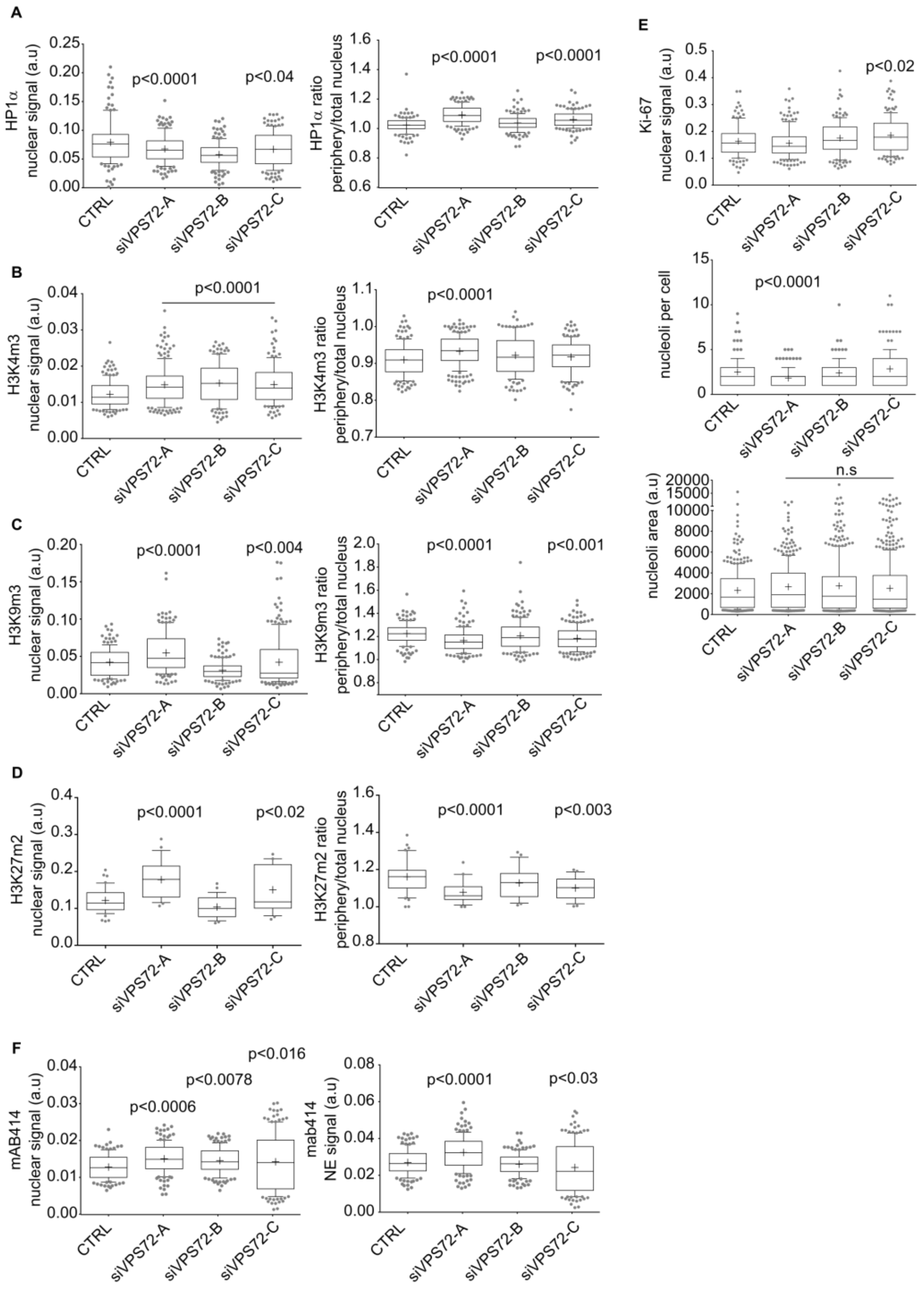

Figure S4. Downregulation of VPS72 does not affect chromatin markers in HeLa Cells. HeLa cells stably expressing mCherry-H2B were transfected with 40 nM siRNA oligos against VPS72. 72 h post transfection cells were fixed and stained with antibodies against the indicated histone modifications **(a-d)**, KI-67 **(e)** and nuclear pore complexes (labeled with mAB414) **(f).** The boxplots (+, indicates mean) correspond to one representative experiment (see materials and methods). **(a-d)** Show mean nuclear intensity and radial distribution of intensity at chromatin periphery. **(e)** Shows the intensity of KI-67 in the nuclear compartment, the number of nucleoli per cell and the nucleoli area. **(f)** Shows nuclear pore marker (mAB414) intensity at nuclear compartment and at nuclear envelope (NE). The data were tested for normality using D'Agostino & Pearson omnibus normality test. When normal distributions could not be assumed, statistical significance at Alpha=0,001 was determined using a Kruskal-Wallis test followed by Dunn´s multiple comparisons test. For normal distributions, ANOVA test followed by Dunnett multiple comparisons test was applied.

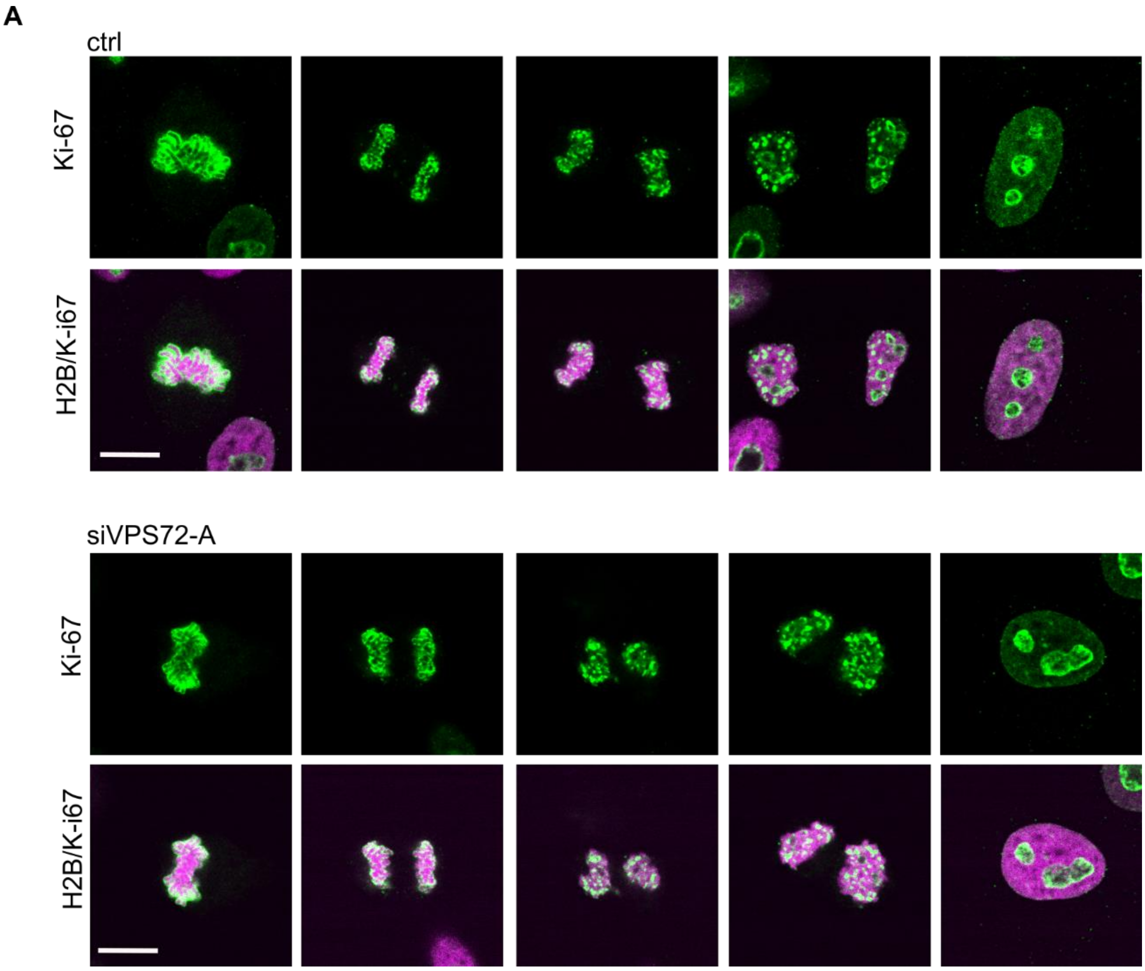

Figure S5. Downregulation of VPS72 does not affect KI-67 distribution during mitotic exit in HeLa cells. (a) HeLa cells expressing mCherry-H2B were transfected with 40 nM control and VPS72 siRNA oligos. 72 h post transfection cells were fixed and stained with KI-67 antibody. Representative confocal images of different mitotic exit time points until interphase are shown. Scale bar 10 μm.

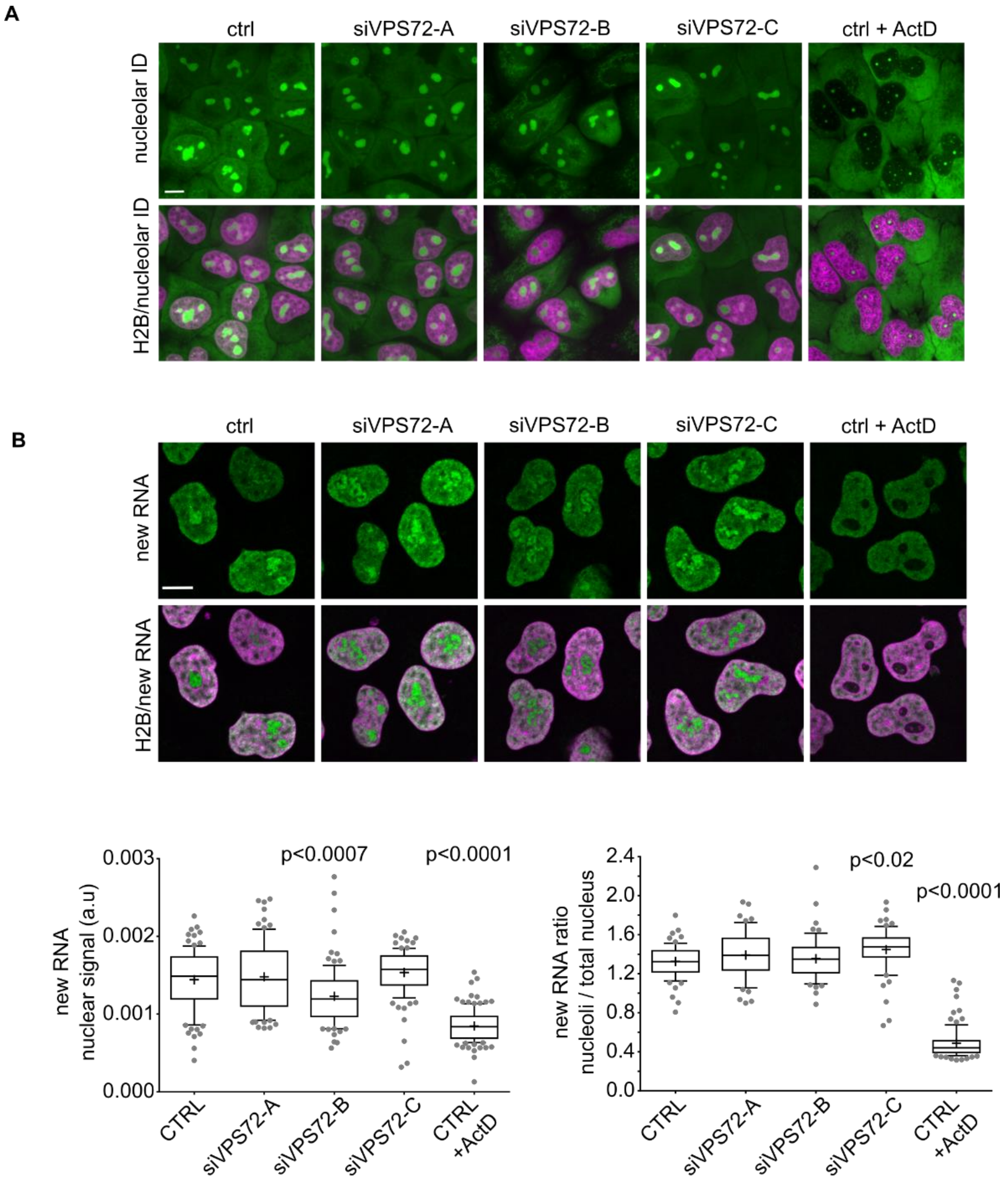

Figure S6. Downregulation of VPS72 does not affect nucleolar integrity and function in HeLa cells. HeLa cells expressing mCherry-H2B were transfected with 40 nM control or indicated VPS72 siRNA oligos for 72 h. **(a)** To check nucleolar integrity living cells were stained with Nucleolar-ID visualize (see materials and methods). As a control, cells were treated with 10 µg/ml Actinomycin D (ActD) for 3 h to induce nucleolar segregation. Scale bar 10 µm. **(b)** Newly synthetized RNA was visualized by uracil incorporation using click-chemistry (see materials and methods). The boxplots (+, indicates mean) show the signal of newly synthetized RNA in the entire nuclear compartment and the ratio of nucleolus vs total nuclear signal. As a control, cells were treated with 50 ng/ml Actinomycin D for 1h to inhibit RNA polymerase I and to abrogate nucleolar RNA synthesis. Scale bar 10 µm.

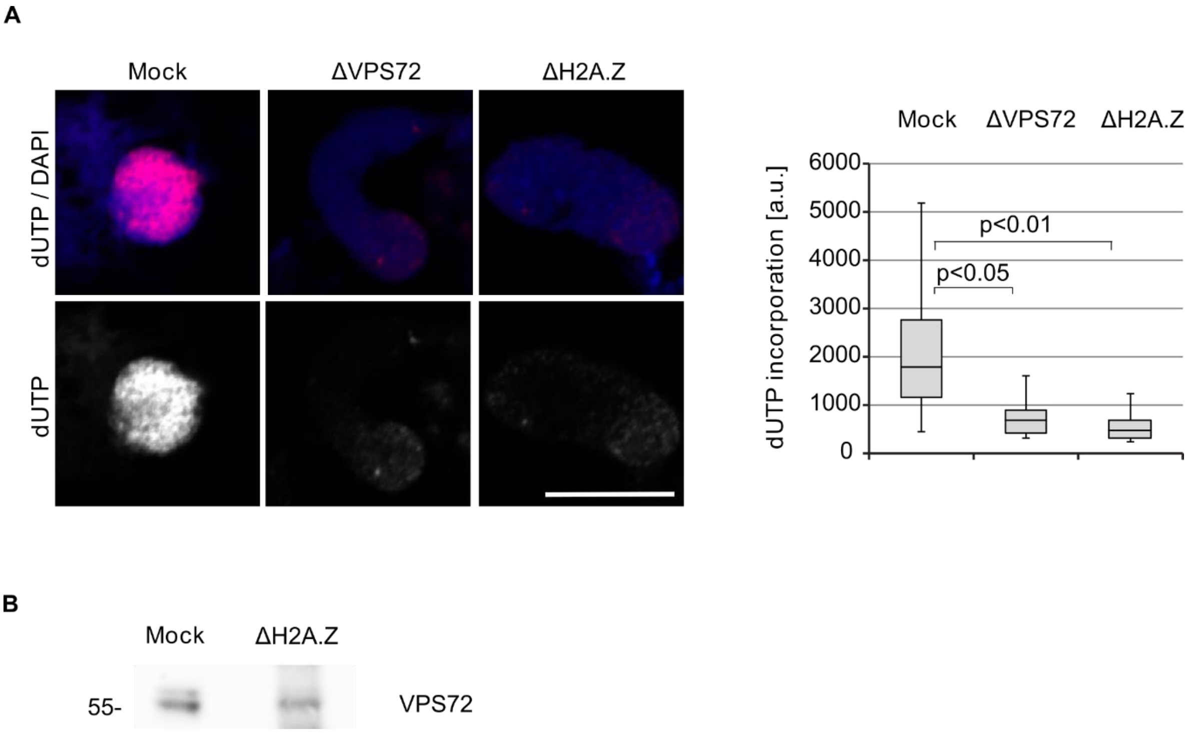

Figure S7. VPS72 or H2A.Z depletion results in DNA replication defects. (a) *Xenopus* egg extract was treated with VPS72 antibody beads or beads coupled to a recombinant N-terminal VPS72 fragment (aa 1-97) to deplete H2A.Z. Each extract was supplemented with 5 μM Cy3-labled dUTP and incubated with sperm for 2 h. The samples were fixed, spun down, and stained with DAPI. Cy3-dUTP intensity on chromatin was quantified. n > 20 structures, N = 2 experiments. Significance values are based on a two-tailed student's test. **(b)** Western blot analysis of VPS72 in egg extracts depleted for H2A.Z.

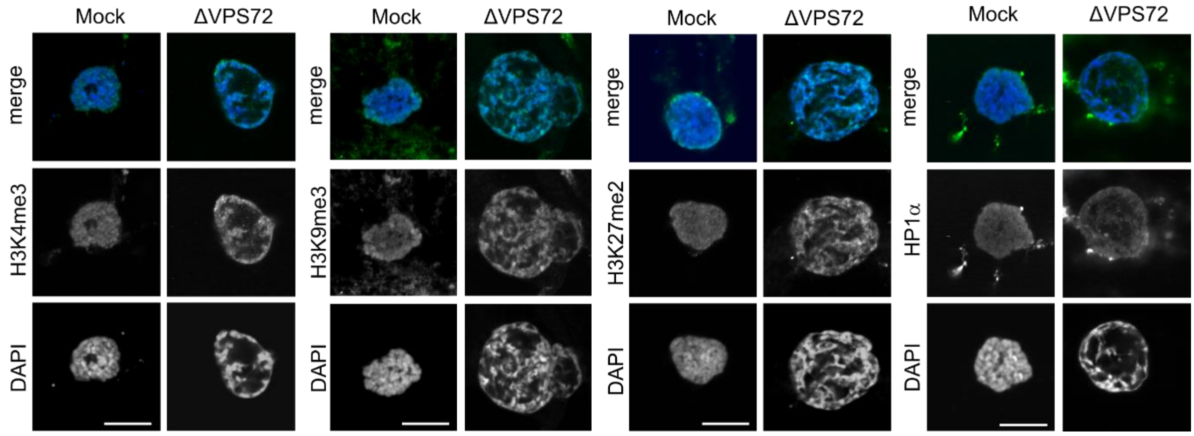

Figure S8. Depletion of VPS72 does not affect chromatin marks on *in vitro* decondensed chromatin. Mitotic chromatin clusters from HeLa cells were incubated in mock or VPS72-depleted egg extracts for 120 min. Samples were fixed with 4% paraformaldehyde and 0.5% glutaraldehyde, stained with DAPI (blue in overlay), and the indicated antibodies and analyzed by confocal microscopy.

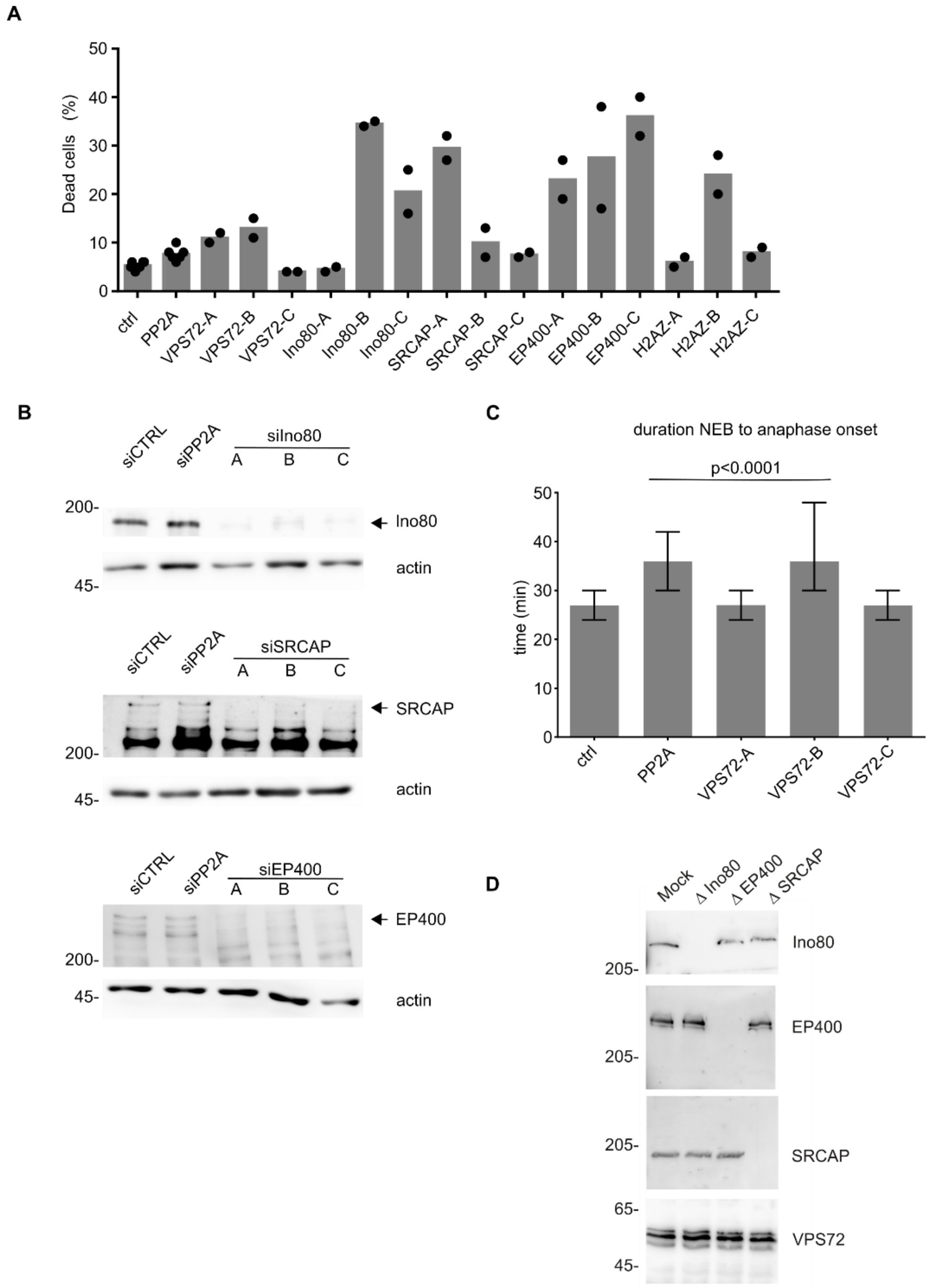

Figure S9. Downregulation of Ino80, SRCAP or EP400 compromises cell viability. **(a)** HeLa cells expressing mCherry-H2B were transfected with 20 nM siRNA oligos against SRCAP, Ino80, EP400, VPS72, PP2A or H2A.Z or a control oligo. 48-96 h after transfection cells were analyzed by life cell imaging. Quantitation shows the percentage of dead cell 90 h post transfection. Bars are the average from the percentage of dead cells from at least two independent experiments (individual data points are indicated). **(b)** Western blot showing the downregulation of Ino80, SRCAP, and EP400 at 72 h post transfection (siRNA 20 nM) in HeLa cells stably expressing mCherry-H2B. **(c)** The median duration of early mitotic progression from nuclear envelope break down (NEB) to anaphase onset is shown for mitotic events analyzed in Figure 1E. Bars represent interquartile range. Statistical significance at Alpha=0,01 was determined using a Kruskal-Wallis test followed by Dunn´s multiple comparisons test. **(d)** Western blot analysis of egg extracts depleted for SRCAP, EP400 or Ino80. Samples were analyzed with indicated antibodies.

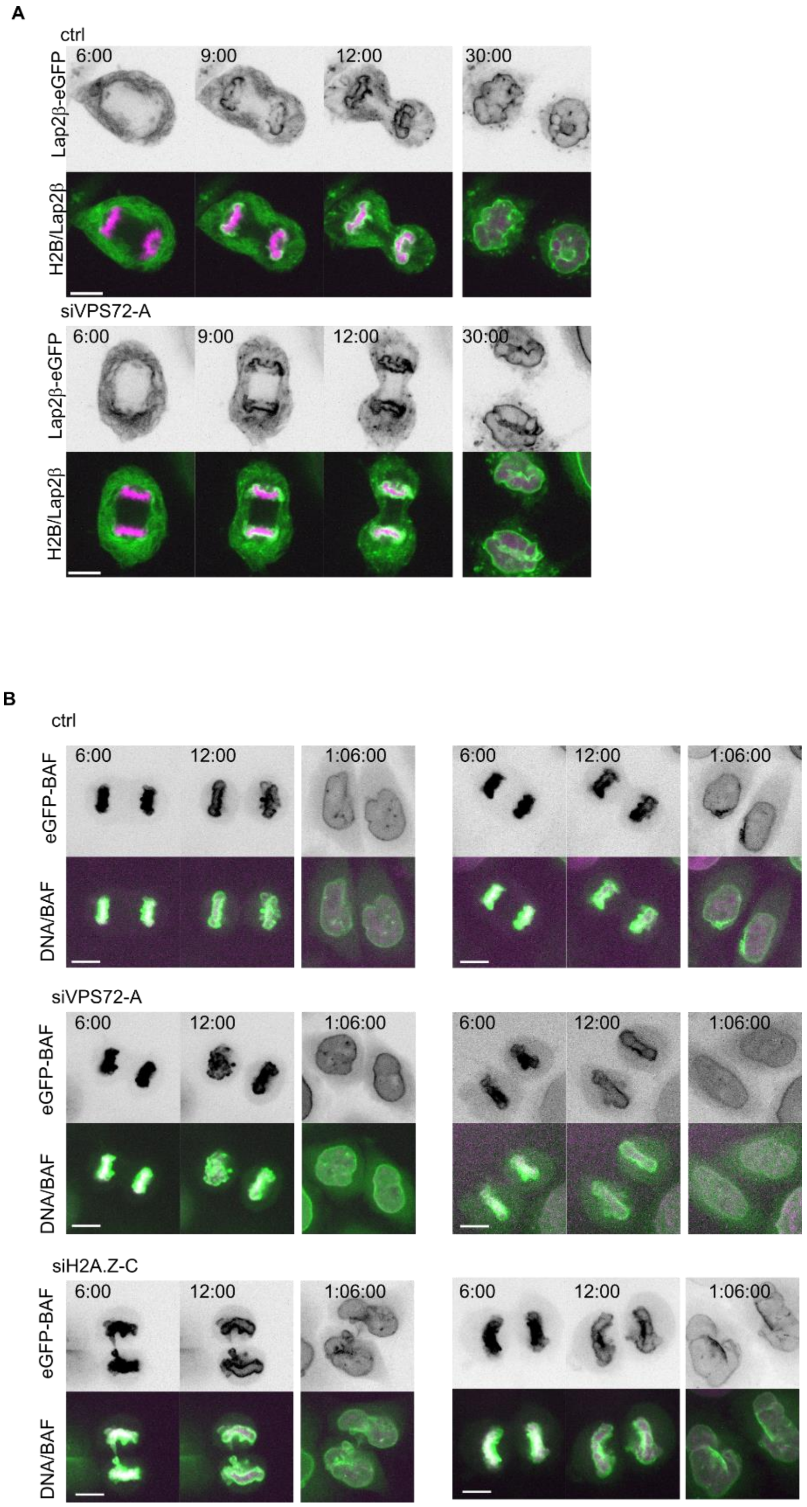

Figure S10. Downregulation of VPS72 does not affect nuclear envelope recruitment during mitotic exit in HeLa cells. (a) HeLa cells expressing mCherry-H2B and Lap2β-EGFP were transfected with 20 nM control and VPS72 siRNA oligos. 72 h post transfection cells were subjected to confocal live-cell imaging. Representative confocal images of different mitotic exit time points are shown. Time is normalized to metaphase to anaphase transition. Scale bar 10 μm. **(b)** HeLa cells expressing BAF-EGFP were transfected with 20 nM control, VPS72 and H2A.Z siRNA oligos. 72 h post transfection cells were incubated in 200 nM Sir-Hoechst and subjected to confocal live-cell imaging. Representative confocal images of different mitotic exit time points are shown. Time is normalized to metaphase to anaphase transition. Scale bar 10 μm.

A

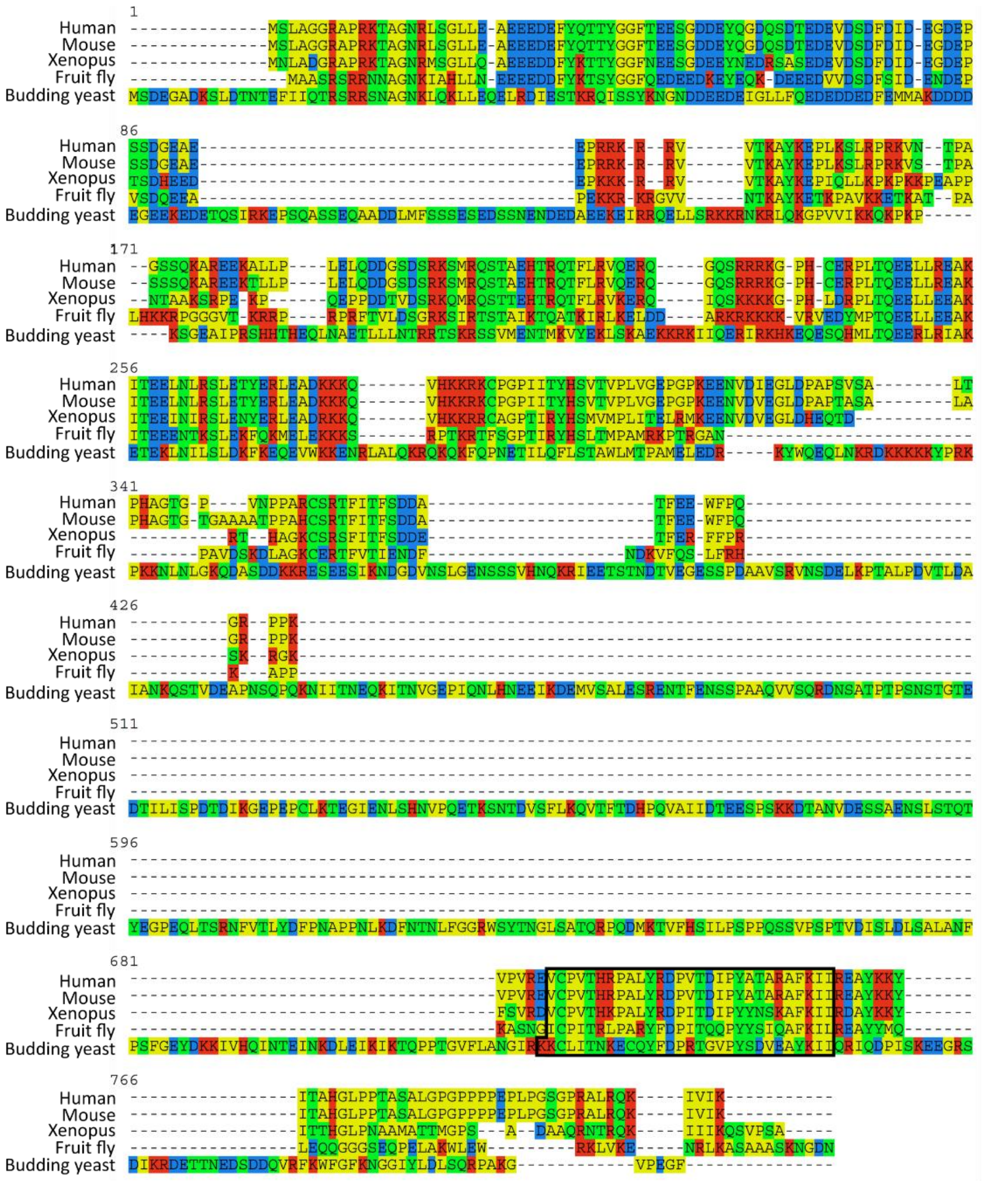

B

Identity score full lenght protein

Human	100%				
Mouse	96.97%	100%			
Xenopus	65.15%	65.72%	100%		
Fruit fly	36.46%	36.18%	38.74%	100%	
Yeast	21.42%	21.19%	22.09%	22.5%	100%
	Human	Mouse	Xenopus	Fruit fly	Yeast

Identity score YL1 domain

Human	100%				
Mouse	100%	100%			
Xenopus	79.31%	79.31%	100%		
Fruit fly	51.72%	51.72%	58.62%	100%	
Yeast	41.37%	41.37%	44.82%	44.82%	100%
	Human	Mouse	Xenopus	Fruit fly	Yeast

Similarity score full lenght protein

Human	100%				
Mouse	97.8%	100%			
Xenopus	74.5%	74.5%	100%		
Fruit fly	46.15%	45.86%	47%	100%	
Yeast	30.76%	30.97%	31.44%	31.9%	100%
	Human	Mouse	Xenopus	Fruit fly	Yeast

Similarity score YL1 domain

Human	100%				
Mouse	100%	100%			
Xenopus	89.65%	89.65%	100%		
Fruit fly	72.41%	72.41%	72.41%	100%	
Yeast	55.17%	55.17%	55.17%	55.17%	100%
	Human	Mouse	Xenopus	Fruit fly	Yeast

Figure S11. Sequence alignment, identity and similarity scores of VPS72 full length and YL-1 domain. (a) Alignment of human, mouse, Xenopus, fruitfly and budding yeast VPS72 proteins. The YL-1-C domains of the proteins, as defined in the NCBI database, are enclosed with a bold black line. **(b)** Pairwise identity and similarity scores of full length VPS72 as well as the YL-1-C domain alone are indicated.
